# A ribonucleotide carbohydrate system (iRNC) enhances antigen presentation and controls glioblastoma

**DOI:** 10.7150/thno.127558

**Published:** 2026-01-14

**Authors:** Hyung Shik Kim, Juhyun Oh, Jueun Jeon, Fan Fei, Sepideh Parvanian, Rainer Kohler, Christopher Garris, Ralph Weissleder

**Affiliations:** 1 Center for Systems Biology, Massachusetts General Hospital, 185 Cambridge St, CPZN 5206, Boston, MA 02114, USA.; 2 Department of Pathology, Massachusetts General Hospital, Boston, MA 02114, USA.; 3 Department of Systems Biology, Harvard Medical School, 200 Longwood Ave, Boston, MA 02115, USA.; 4 Graduate School for Biomedical Science & Engineering, Hanyang University, 04763, Seoul, Korea.

**Keywords:** mRNA, nanoparticles, targeting, vaccine, dendritic cells

## Abstract

**Rationale:** Messenger RNA (mRNA)-based cancer vaccines hold great potential as immunotherapeutic agents; however, their clinical translation remains hindered by inefficient systemic delivery, suboptimal antigen presentation, and formulation-associated toxicity of lipid nanoparticles (LNPs). To address these issues, we sought to design a delivery platform that couples antigen expression and innate immune stimulation within a single nanostructure.

**Methods**: We engineered a synthetic immuno-ribonucleocarbohydrate (iRNC) system constructed from fluorinated cyclodextrin nanoparticles. This modular platform co-delivers mRNA and small-molecule NFκB agonists, enabling simultaneous antigen expression and immune activation. Using ovalbumin (OVA) mRNA as a model antigen, we evaluated biodistribution, immune activation, and therapeutic efficacy in CT2A orthotopic glioblastoma models following systemic administration.

**Results**: iRNCs were preferentially internalized by tumor-associated phagocytes, leading to efficient mRNA transfection and antigen presentation within the glioblastoma microenvironment. This dual-function system elicited robust innate immune activation with minimal systemic toxicity. Importantly, iRNC vaccination demonstrated both prophylactic and therapeutic efficacy in CT2A-bearing mice, significantly suppressing tumor growth and extending survival compared to conventional LNP formulations.

**Conclusion**: The iRNC platform unifies mRNA delivery and immune stimulation into a single, programmable nanoparticle, providing a distinct and clinically relevant strategy for systemic mRNA vaccination. Its ability to reprogram tumor-associated phagocytes and induce potent anti-tumor immunity underscores its promise as a next-generation platform for cancer immunotherapy.

## Introduction

Advancements in nanotechnology and mRNA engineering have paved the way for more efficient vaccines, including tumor vaccines. Lipid nanoparticles (LNP) of various compositions^1^, ^2^ and immunostimulatory polymers^3^ have been utilized as delivery systems and, on occasion, as adjuvants for immunotherapy^4^. Alternatively, more efficient designs have drawn inspiration from virus nanoparticles, such as multilamellar vesicles^5^, virus-like particles^6^, particles in particles^7^ among others^8^, ^9^. Despite these advancements, there remains room for improvement, as many materials still exhibit low efficiency (both in delivering to target cells and transfecting them with mRNA payloads), while toxicity (components and dose) remains a concern. More efficient future systems will necessitate the following improvements: i) enhanced delivery to target cells, specifically antigen presenting cells such as macrophages and dendritic cells, ii) improved transfection of mRNA payloads, iii) incorporated adjuvants for optimal local immune stimulation, and iv) minimal off-target toxicity.

To elicit a robust immune response, antigens must be effectively presented by antigen-presenting cells (APCs) to T-cells. Our research group and others have been interested in more efficient dendritic cell (DC)-targeting approaches and have focused on carbohydrate complexes that are naturally recognized by phagocytic cells^10^. Recently, we have demonstrated that nanoparticle-targeted phagocytic cells can be induced into immunostimulatory phenotypes^11^-^13^. These nanoparticulate carbohydrate materials (CANDI) were based on modified lysine crosslinked bis-succinyl cyclodextrins for guest-host chemistry. We have also shown that transfection efficiency can be significantly enhanced by fluorination of carbohydrate constructs (RNC^14^). Prior advancements were primarily used to repolarize macrophages from immunosuppressive to immunostimulatory phenotypes^15^ or to transfect cells with green fluorescent protein (GFP)^14^. However, it was unknown whether the RNC platform could be developed for innate immunotherapy or cancer vaccination, what the optimal components were, and how the composite material would compare to other nanosystems for mRNA delivery. Through further design modifications and optimizations, we present evidence that immunomodulatory ribonucleocarbohydrate complexes (iRNC) exhibit significantly higher efficiency than LNP in stimulating DC and fostering highly effective antitumor responses. The optimized cancer vaccine elicits potent T-cell recruitment into glioblastoma and achieves complete tumor control.

## Results

### Synthesis and design of efficient delivery and myeloid activation system

Tumor antigens are presented to both CD4+ and CD8+ T cells by professional antigen-presenting cells (pAPCs) using major histocompatibility complexes (MHC) I and II respectively. Tumor antigen cross-presentation by type 1 conventional dendritic cells (cDC1), in particular, is thought to be crucial for antitumor CD8+ T cell responses. Nevertheless, emerging studies reveal that within the tumor microenvironment, several non-APC populations can likewise present tumor antigens through both MHC class I and II pathways. These cells, occasionally referred to as **“**amateur” APCs (aAPCs), include tumor-associated macrophages^16^, fibroblasts^17^, and even cancer cells^18^, all of which are far more abundant than pAPCs in the TME and may substantially play a significant role in antitumor immune response. This is all the more important as i) cDC1 are scant^19^, and ii) tumors exploit multiple escape mechanisms to evade immune recognition^20^. Thus, broadly targeting more abundant phagocytic cells in the TME is a strategy to enhance antigen presentation. This is particularly important for immune deserted and difficult-to-treat tumors such as glioblastoma.

**Figure 1:**
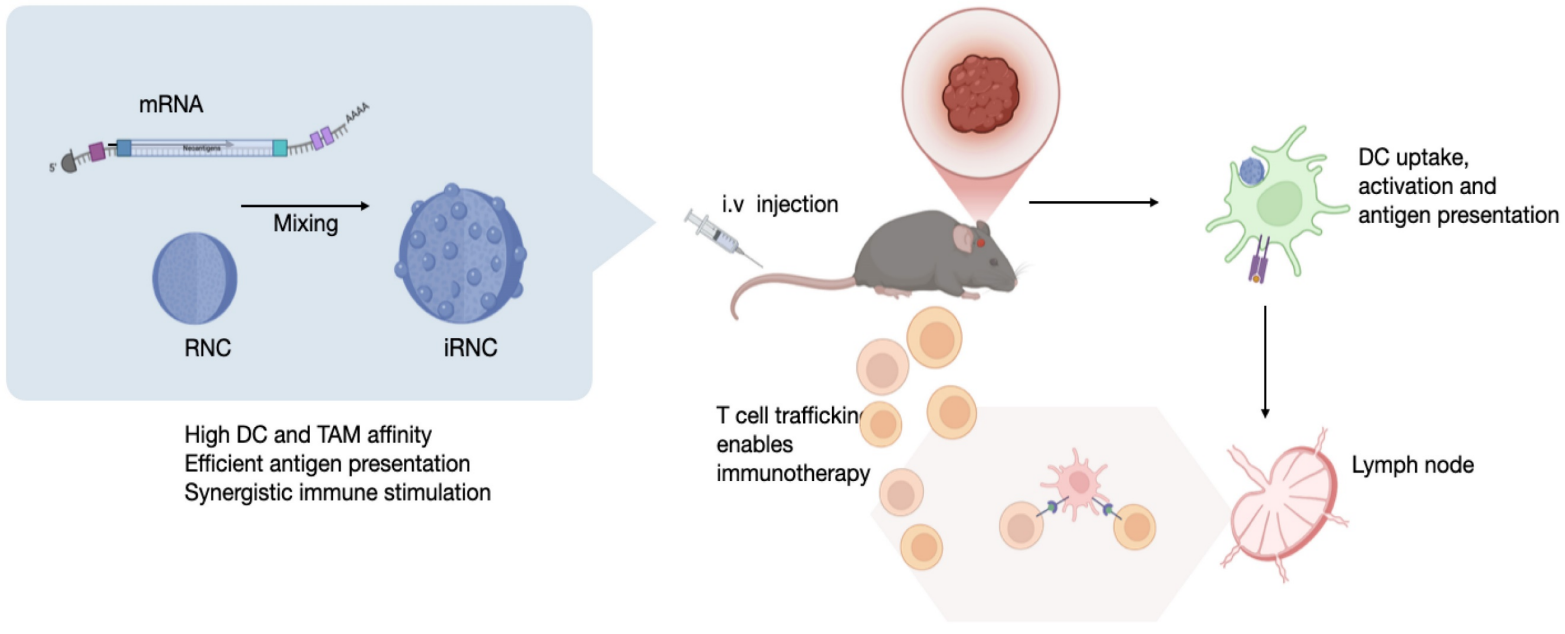
** Overview of the myeloid cell immunostimulatory mRNA delivery platform. (A).** The ImmunoRNC system is composed of perfluorocarbon-functionalized cyclodextrin nanoparticles, which are loaded with a combination of TLR agonists and NF-kB stimulants. Upon condensation with mRNA, the nanoparticles undergo compaction, resulting in a compact structure with single-unit clustering that increases in size to approximately 200 nm (iRNC). **(B).** Following systemic injection, iRNCs efficiently accumulate in tumor-associated macrophages (TAMs) and dendritic cells (DCs), which are often immunosuppressive in the tumor microenvironment. The small-molecule NF-kB modulators within the nanoparticles activate these cells, promoting immune activation. Additionally, this demonstrates high *in vivo* transfection efficiency of mRNA in myeloid cells, which can lead to robust anti-tumor immune responses and therapeutic efficacy as was tested in the OVA glioblastoma model in this study.

Carbohydrate polymers and nanoparticles have been used to efficiently target phagocytic cells in the TME^10^, given their favorable pharmacokinetics^21^, recognition to be internalized and processed by the cellular machinery, and ability to influence the cellular phenotype by pathway modulation^15^. Based on prior insights into targeting and reprogramming of tumor-associated myeloid cells^11^, ^12^, ^22^ we used a poly-fluorinated cyclodextrin scaffold with cationic crosslinkers (**Figure 1**) to condense mRNA into ~150-200 nm immuno ribonucleocarbohydrate constructs (iRNC). To benchmark the immunotherapeutic potential of this system, we employed full-length ovalbumin (OVA) mRNA as a model tumor neoantigen, a widely accepted system for evaluating antigen-specific responses^23^. The resulting optimized formulation, denoted RNC734, represents an integrated, modular nanoplatform capable of co-delivering nucleic acids and small-molecule immune modulators with precision to tumor-associated phagocytes. This design offers a significant advance in mRNA vaccine delivery by unifying antigen presentation and immune stimulation within a single, tunable construct.

### Characterization of nanocomplexes

**Figure 2:**
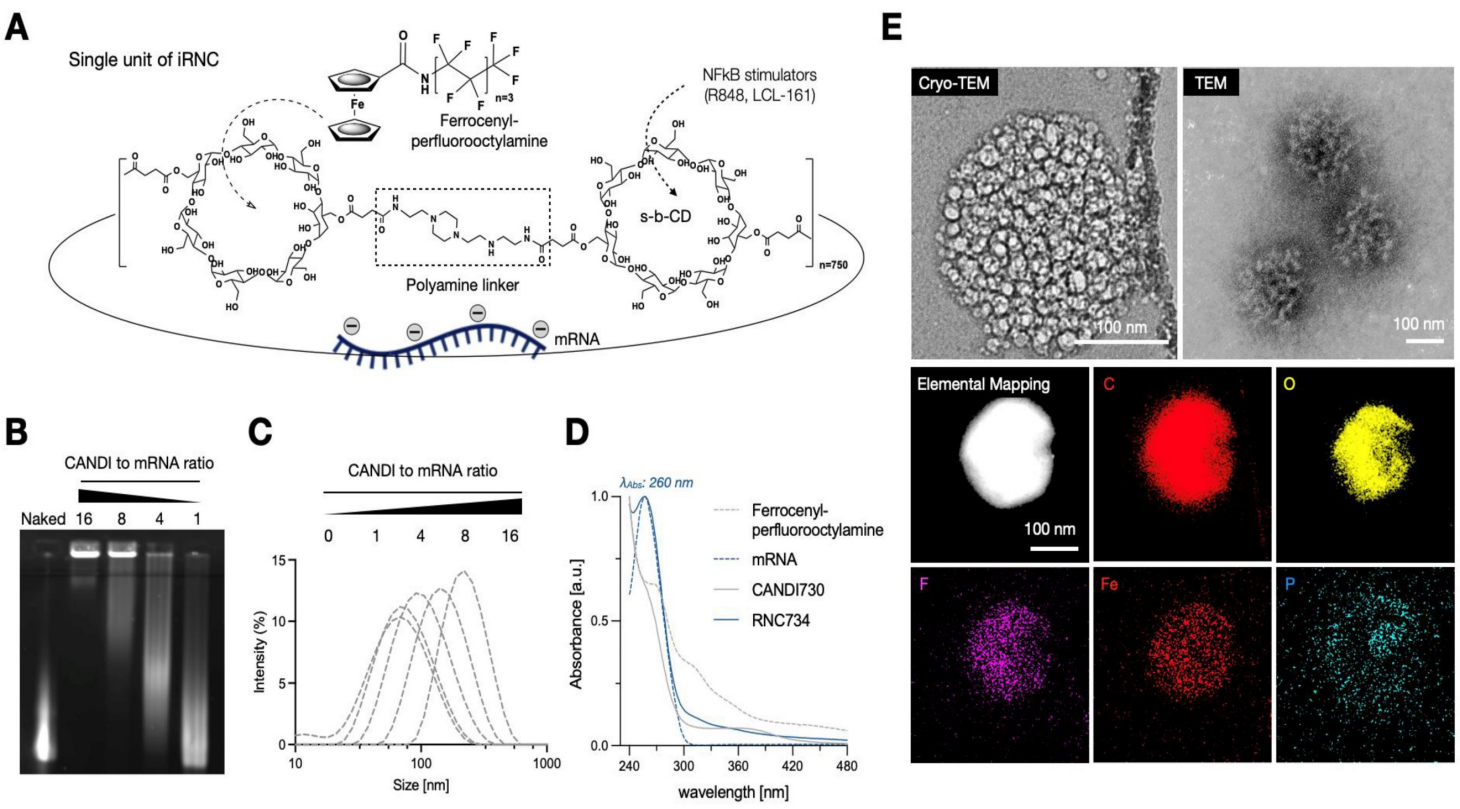
** Characterization of iRNC. (A).** Schematic representation of a prototypical iRNC composition. The nanomaterial is made up of cross-linked cyclodextrin units that serve as guest host reservoirs for NFkB stimulators. The polyamine cross linker (NAPED) allows mRNA condensation (OVA mRNA in this study) while the poly-fluorinated ferrocenyl serves to enhance transfection efficiency. **(B).** Gel retardation assay confirming efficient mRNA encapsulation and stabilization at varying N/P ratios.** (C).** Dynamic light scattering (DLS) analysis illustrating size optimization as a function of the N/P ratio, with a progression toward better condensation at higher ratios. **(D).** UV-Vis spectra of individual components of iRNC. The spectra show distinct absorption peaks corresponding to each component: mRNA exhibits a characteristic peak at 260 nm, LCL161 shows a broad absorbance around 265-280 nm, and R848 absorbs maximally at 328 nm. In addition, a broad metal-to-ligand charge transfer (MLCT) band between 300-500 nm is observed, attributed to the ferrocene moiety introduced via ferrocenyl perfluorooctylamine incorporation into the complex. **(E).** Cryo-TEM, conventional TEM, and EDS mapping images illustrate the nanoscale architecture of the mRNA-condensed RNC-734 formulation. The formulation exhibits a uniform morphology with an average size of approximately 200 nm, showing well-dispersed individual components.

**Figure 2A** summarizes the iRNC components for most efficient antigen presentation, targeting, and general *in vivo* use including: i) bis succinyl cyclodextrin scaffold with cationic polyamine crosslinkers (NAPED) consisting of roughly 1,500 CD units, ii) host-guest incorporated ferrocenyl perfluorooctylamine to enhance transfection efficiency^14^, iii) host-guest incorporated small molecules for NFkB pathway stimulation (R848, LCL-161) and iv) full-length OVA mRNA charge condensed via the polyamine linkers. To determine the optimum mRNA/RNC loading capacity we created different ratio preparations and investigated them by gel electrophoresis. We determined experimentally that a 16:1 ratio (w/w of RNC/mRNA) resulted in compact (**Figure 2B**), uniform morphology with an average size of approximately 200 nm (PDI 0.13). The overall size of different RNC batches was consistent and reproducible, ranging < 1% in diameter (**Figure 2C**). Co-loading of small molecules and mRNA into single nanoparticles was confirmed, and their release profiles were characterized over time (**Figure S2**). Because the amounts of R848 and LCL161 used in the formulation were well below the maximal loading capacity of the CANDI scaffold (0.1 mg per mg CANDI), both small-molecule components were fully incorporated during assembly, resulting in effectively complete loading under these conditions. UV-visible spectra confirmed the successful incorporation of each component within the iRNC complex (**Figure 2D**). These findings were further corroborated by structural analyses. By TEM, RNC734 appeared as homogeneous, spherical nanoparticles of approximately 200 nm in diameter. To further determine the internal architecture and condensation of the complexes, we performed cryoEM, which identified smaller RNC subunits that assemble into the higher-order RNC734^Hi^ structure. The detailed size distribution and surface charge of these subunits are summarized in **Table 1**. These structural observations were additionally supported by elemental mapping (**Figure 2E**).

### Comparative efficacy in myeloid-cell transfection, stimulation and antigen presentation

**Figure 3:**
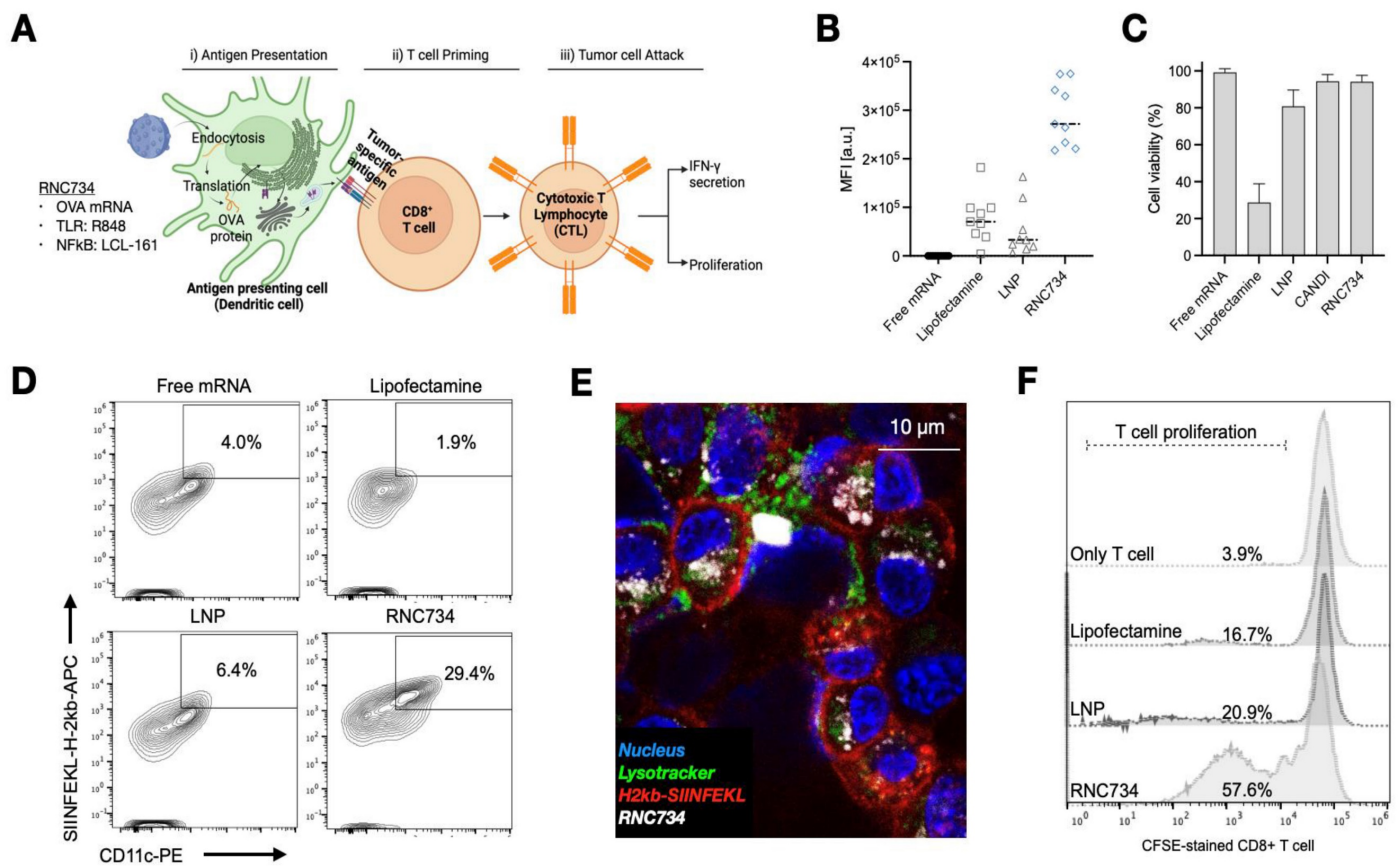
** Cellular effects of RNC734. (A).** Schematic illustration of the RNC734 mechanism. OVA mRNA delivery via RNC734 induces antigen presentation in dendritic cells (DCs), leading to the priming of CD8+ T cells, which differentiate into cytotoxic T lymphocytes (CTLs) capable of killing tumor cells.** (B).** Transfection efficiency of different mRNA constructs in macrophages. mRNA was given in free form or encapsulated in lipofectamine, LNP or RNC. Note the much higher mRNA transfection with RNC compared to LNP or lipofectamine. **(C).** Evaluation of cell viability across different mRNA formulations. Note the cytotoxicity of lipofectamine and LNP. **(D).** Representative flow cytometry plot demonstrating the presentation of the SIINFEKL-H-2Kb complex on bone marrow-derived dendritic cells (BMDCs), confirming effective antigen presentation. **(E).** Confocal microscope image of BMDC cells traced with RNC734 (white) and stained with H2kb SIINFEKL antibody (red), lysotracker (green), and Hoechst33342 (blue). See **Figure. S4** for additional images. **(F).** OT-1 CD8+ T cell expansion was measured after co-culture of T cells with pretreated BMDCs for 72 h using the carboxyfluorescein succinimidyl ester (CFSE) dilution method. The BMDC were exposed to lipofectamine, LNP or RNC734 for 24 h. RNC734 showed the highest OT-1 CD8+ T proliferation.

To evaluate the performance of RNC734 in APCs, we conducted a series of *in vitro* experiments focusing on APCs transfection, activation, and subsequent T cell stimulation (**Figure 3**). A secondary goal was to determine how efficient the RNC734 system was compared to naked mRNA, lipofectamine and LNP-mRNA, commonly used platforms for mRNA delivery. We first assessed cellular uptake in immortalized murine bone marrow-derived macrophages (iMACs) using fluorescently labeled RNC734 (**Figure S3**). The data show that up to 100% of incubated cells could be labeled (at concentrations above 1 mg/mL) but still did not show any appreciable cellular toxicity. Confocal microscopy at 24 h post-treatment indicated lysosomal escape of the RNC734, and importantly, surface presentation of SIINFEKL peptide on MHC I molecules was clearly detected (**Figure S4**). We next performed side-by-side transfection comparisons using GFP mRNA delivered by RNC734, lipofectamine, LNP, and naked mRNA. RNC734 demonstrated the highest transfection performance, showing approximately a 5-fold increase in mean fluorescence intensity (MFI) compared with LNP (**Figure 3B**). Cell viability assays further confirmed the safety of iRNCs, showing minimal cytotoxicity (<2%) compared to lipofectamine (71.3%) and LNP (19.2%) (**Figure 3C**).

**Figure 4:**
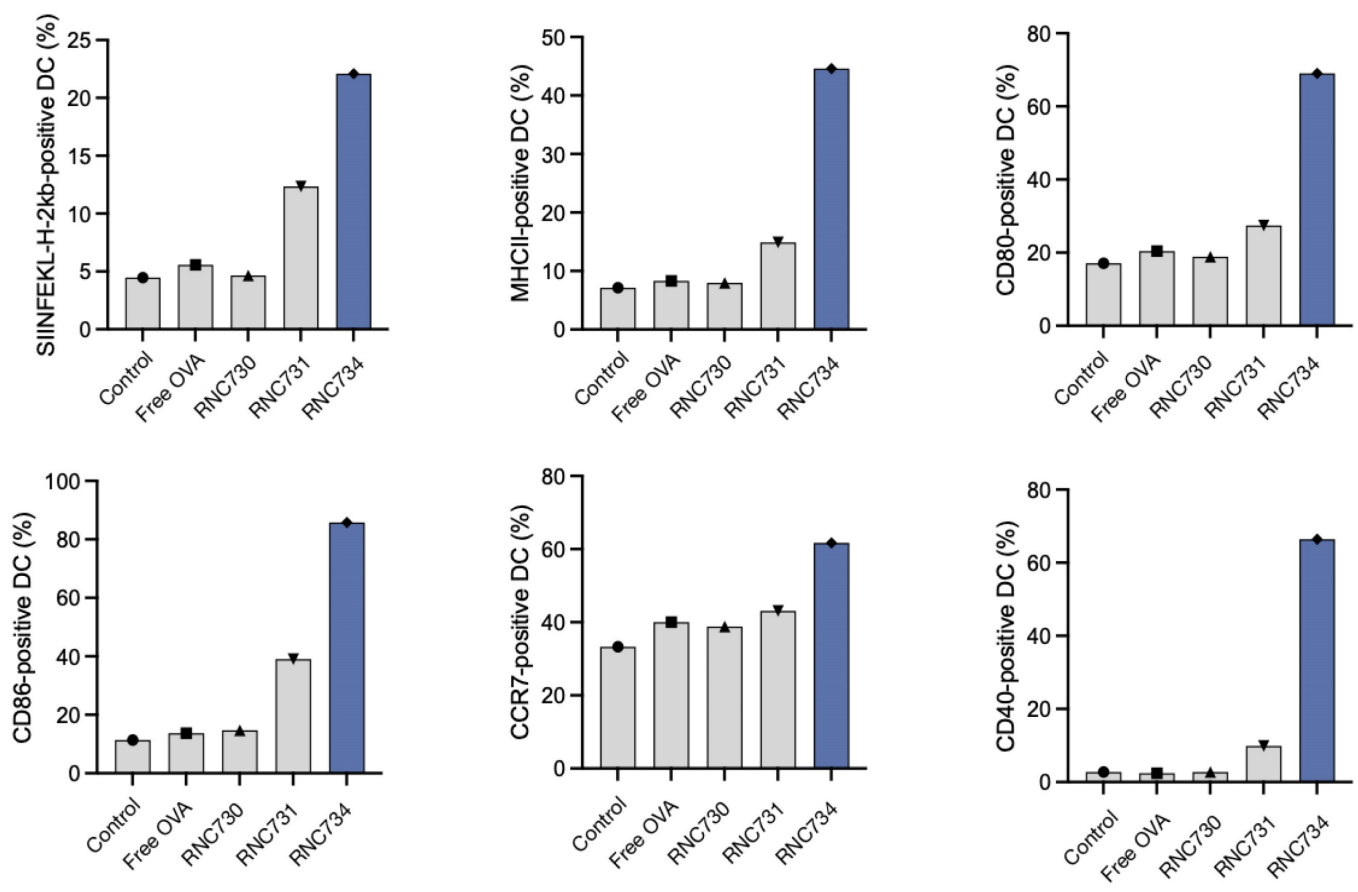
** Dendritic cell activation following RNC exposure.** BMDC isolated from BL6 donor mice were exposed to RNC734, RNC730, RNC731 free OVA mRNA or control PBS. Panels A-F show different activation markers identified in the different treatment groups. Note the enhanced activation status and antigen presentation ability of BMDC upon treatment with RNC734. The compositions of RNC730, RNC731, and RNC734 are detailed in **Table 1.**

To investigate functional antigen presentation, bone marrow-derived DCs were incubated with different mRNA formulations, and expression of the SIINFEKL-H-2Kb complex was measured on dendritic cells. RNC734 treatment resulted in the highest proportion of antigen presenting DCs (29.4% of cells) significantly outperforming LNP-mRNA (6.4%; p <0.001), lipofectamine (1.9%) or naked mRNA (4%) (**Figure 3D** and **3E**). In parallel, we determined naïve OT-1 CD8+ T cell expansion after co-culture of T cells with mRNA pre-treated DCs. Again, RNC734 showed higher T-cell proliferation (57.6%) compared to LNP-mRNA (20.9%; p < 0.01) or lipofectamine-mRNA (16.7%; p < 0.01) (**Figure 3F**). Futhermore, RNC734-treated DCs showed robust upregulation of activation markers including MHCII, CD80, CD86, CCR7, and CD40, indicating effective DC maturation (**Figure 4**). Supporting experiments confirmed increased interferon-ɣ production by T cells in co-cultures (**Figure S5**), and proliferation of T cells in co-cultures (**Figure S6**), reinforcing the superior immune stimulatory effect of RNC734 over conventional mRNA delivery preparations.

### *In vivo* iRNC distribution upon systemic administration

**Figure 5:**
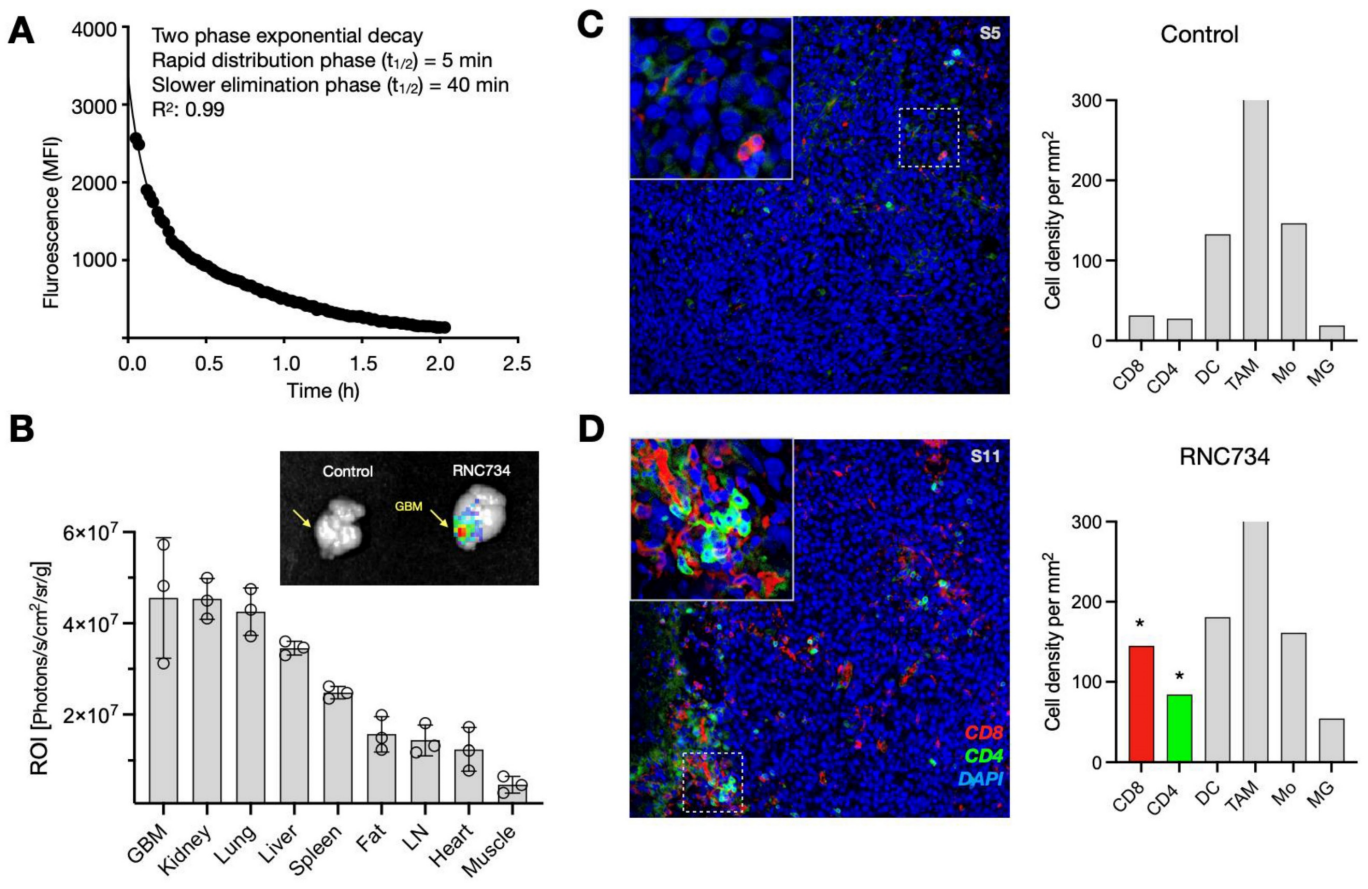
** RNC734 pharmacokinetics and cellular effects. (A).** Blood half-life of fluorescently labeled RNC734 (RNC734-AF647), determined via serial non-invasive imaging of capillaries in a live mouse. The nanoparticles exhibit a biphasic exponential decay profile following intravenous injection, with a fast half-life (T_1/2_: 4 min) and a slow half-life (T_1/2_: 40 min), consistent with nanoparticle-like behavior. **(B).** Organ biodistribution following systemic administration. Mice bearing intracranial CT2A glioblastoma received an intravenous dose of 5 mg RNC734-AF647, and organs were collected 12 h post-injection. Note the high accumulation of RNC734 in glioblastoma tissue but also in other tissues with RES (liver, spleen) or elimination routes (kidneys). **(C-D).** Immunohistochemistry of orthotopic glioblastoma in PBS control **(C)** or RNC734 **(D)** treated mouse. RNC734-treated mice showed significantly higher CD8 and CD4 levels. See **Figure. S10** for additional stains.

Systemic administration is increasingly favored for cancer vaccines, as it allows for broader targeting of disseminated or metastatic lesions compared to localized intratumoral injection. Moreover, systemic vaccination has been shown to elicit stronger T cell responses^24^, ^25^, and offers practical advantages by eliminating the need for image-guided delivery and enabling consistent dosing across heterogeneous tumor sites. For systemic approaches to be effective, however, mRNA-loaded nanoparticles must achieve sufficient circulation time and preferential accumulation within the tumor microenvironment. To explore the pharmacokinetics, we used intravital imaging to first determine the blood half-life of AF647-labeled RNC734. We observed a biphasic decay pattern consisting of a slow phase (40 min; 0.66 h) and a rapid phase (5 min; 0.075 h) with a good curve fit (R^2^: 0.99; **Figure 5A**). Despite the high cardiac output and metabolic rate in mice, such extended half-life suggests favorable systemic retention suitable for tumor targeting.

Biodistribution experiments of fluorescently labeled RNC734 showed predominant accumulation in orthotopic glioblastoma tissue, as well as in kidney, lung, liver spleen, and lymph nodes (**Figure 5B** and **Figure S7**). Fluorescent intensities were quantified and normalized to each of tissue weight to ensure accurate comparison across organs. Notably, accumulation in glioblastoma was facilitated by the leaky vasculature and compromised blood-brain barrier (BBB), characteristic of high-grade gliomas. Furthermore, the iRNC**'**s moderate size (~200 nm), mild cationic surface charge, and fluorinated host-guest chemistry are likely to have contributed to enhanced transport across the BBB^26^ and selective retention within the tumor microenvironment.

Flow cytometry confirmed that the majority of RNC734 localized within tumor-associated phagocytes, specifically CD11b+ F4/80+ macrophages and CD11c+, MHCII+ dendritic cells (**Figure S8**) with much lower uptake by other cells. To further validate this selective targeting, we employed the ROSA26 Cre-inducible GFP mice model, in which Cre mRNA delivered via RNC734 induced GFP expression specifically in tumor-infiltrating phagocytes. These results confirmed that functional transfection was largely restricted to myeloid antigen-presenting cells within the tumor microenvironment (**Figure S9**). Consistent with these findings, histological analysis of brain tumors in the orthotopic glioblastoma model demonstrated markedly increased immune cell infiltration in RNC734-treated mice, particularly CD8⁺ and CD4⁺ T cells (**Figure 5C-D** and **Figure S10**), supporting the role of RNC734 in effective immunomodulation within the brain tumor microenvironment.

### Efficacy in treating glioblastoma

**Figure 6:**
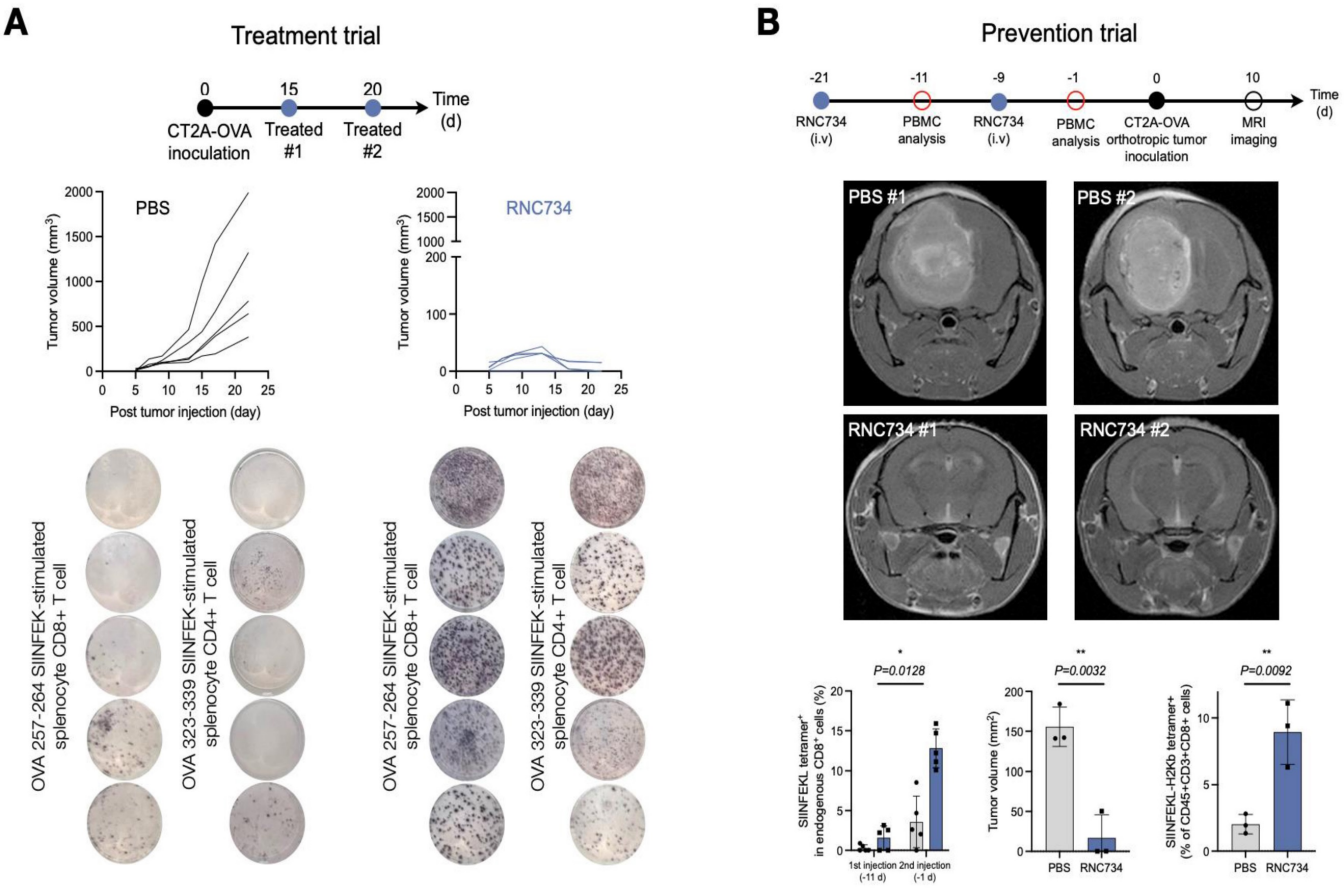
** RNC boosts myeloid-targeted mRNA vaccination and reprograms the immunosuppressive tumor microenvironment in the CT2a-GBM model. (A). ImmunoRNC drives potent anti-tumor immunity.** Tumor growth curves show significant inhibition of tumor progression in RNC734-treated mice. IFNγ ELISpot analysis of blood cells collected 25 days post-treatment reveals a striking increase in CD8⁺ and CD4⁺ T-cell responses, indicated by intense purple staining in RNC734-treated groups. **B. Prophylactic vaccination reshapes glioblastoma immunity.** Schematic of the preventive treatment strategy in the orthotopic CT2a-GBM model, where mice received intravenous (i.v.) RNC734 or PBS. Representative MRI images (day 10 after implantation) demonstrate significantly reduced GBM tumor volumes (p = 0.0032) in RNC734-treated mice. Flow cytometry analysis of peripheral blood mononuclear cells (PBMCs) shows a marked increase in SIINFEKL peptide tetramer-positive CD8⁺ T cells after the second RNC734 injection. Tumor mass quantification (p = 0.0128) and analysis of tetramer-positive T cells within the TME (p = 0.0092) confirm a robust anti-tumor immune response, highlighting the therapeutic potential of myeloid-targeted mRNA vaccination in glioblastoma.

We next set out to test the therapeutic efficacy of RNC734 in the orthotopic CT2A glioblastoma model. We performed two different trials: a treatment trial (**Figure 6A**) and a prevention trial (**Figure 6B**). In the treatment trial, we implanted CT2A-OVA tumor cells and performed treatment with RNC734 or PBS 15 and 20 days later. As expected, there was exponential tumor growth in the PBS-treated animals (mean tumor volume on day 22: 1000 ± 200 mm^3^). However, with RNC734 treatment, the tumors uniformly decreased in size (mean tumor volume on day 22: 10 ± 20 mm^3^) and were eventually eliminated in 4/5 animals. To further assess vaccine-induced antigen-specific T cell responses, we collected peripheral blood mononuclear cells (PBMCs) on day 25 and performed an IFN-γ ELISPOT assays following ex vivo re-stimulation with class I (SIINFEKL) and class II (ISQAVHAAHAEINEAGR) OVA peptides. Mice treated with RNC734 exhibited significantly elevated numbers of IFN-γ-secreting CD8⁺ and CD4⁺ T cells, respectively, compared to PBS controls. These findings indicate that the RNC734 vaccination elicited a broad and functional T cell response, encompassing both cytotoxic and helper subsets, which supports its protective efficacy against glioblastoma recurrence.

In a second experiment, we attempted to answer whether prophylactic administration of RNC734 could confer protective immunity against subsequent tumor challenge, which is a clinically relevant scenario in individuals at high risk for glioblastoma recurrence, such as those exposed to cranial irradiation. Mice received two intravenous administrations of RNC734, followed seven days later by intracranial implantation of CT2A-OVA tumor cells. Tumor progression was monitored by serial MRI. Animals sham treated with PBS developed large intracranial tumors (mean tumor volume on day 10: 100 ± 20 mm^3^) whereas RNC734 vaccinated animals had no or very small tumors (mean tumor volume on day 10: 10 ± 2 mm^3^; **Figure 6B**). Flow cytometry analysis of PBMCs exhibited a robust expansion of SIINFEKL tetramer-positive CD8+ T cells following the second RNC734 injection. These findings suggest that RNC734 induces durable cellular immunity capable of preventing glioblastoma establishment in high-risk settings.

**Figure 7:**
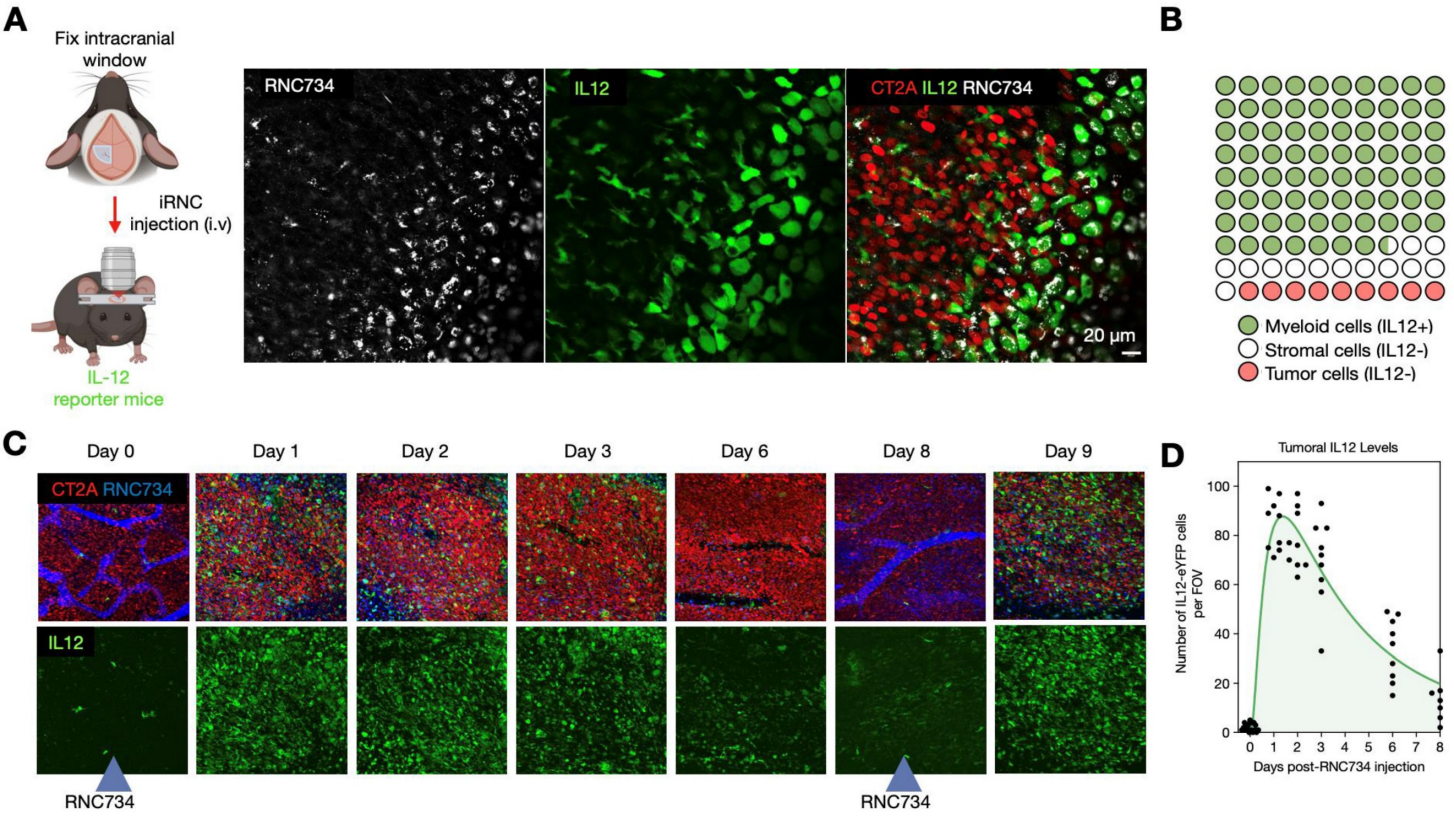
** Kinetics of cellular RNC734 accumulation and immune stimulation**. **(A).** Intravital imaging of iRNCs kinetics in the orthotropic CT2A tumor (CT2A-H2B-apple, red) microenvironment in IL12-eYFP reporter mice (green). Systemically administered RNC734 (white) is imaged 24 h after injection and seen in puncta within cells. **(B).** The vast majority of RNC734-positive cells produce high amounts of IL12 and are myeloid cells. A smaller fraction accumulates in non-IL12-producing stromal cells. **(C)**. Serial in vivo imaging of the tumor microenvironment in a brain window chamber. Note the strong IL12 induction (green cells) within 24 h after IV administration of RNC734. Levels decrease over 6-8 days but can be restored to high levels with a repeat RNC734 injection. **(D)**. Temporal analysis of IL12 induction in the TME with a single IV injection of RNC734 on day 0. See **Figure S12** for statistics.

To further elucidate the mechanism underlying the therapeutic efficacy of iRNC in glioblastoma, we performed intravital imaging using a brain tumor model^27^-^29^. IL-12 eYFP reporter mice were implanted with CT2A-H2B-apple glioma cells, and the pharmacokinetics and single cell dynamics of AF647 labeled-RNC734 were assessed over time. Within 24 h after IV administration, RNC734 was observed within non-tumor stromal cells in the tumor microenvironment, which exhibited robust IL-12 expression (**Figure 7A**). In contrast, response was absent in control animals receiving payload-free RNCs. Quantitative image analysis showed that over 90% of IL-12-producing cells contained RNC734 puncta (**Figure 7B** and **Figure S11**), consistent with *in vitro* uptake patterns (**Figure S4**). Temporal profiling revealed that IL-12 expression peaked within one day and declined by days 6-8. Notably, a second systemic dose of RNC734 administered at this time point successfully re-induced high levels of tumoral IL-12 (**Figure 7C-**7**D** and **Figure S12**). Together, these data demonstrate that systemically delivered RNC734 selectively engages tumor-associated immune cells, induces IL-12 production, and contributes to sustained anti-tumor immune activation.

## Discussion

High-grade glioblastoma represent the most prevalent and lethal primary malignant tumor arising in the central nervous system. Standard-of-care (SOC) therapies include surgical resection with chemoradiation and other FDA-approved treatments such as intracavitary chemotherapy wafers, bevacizumab, and alternating electrical fields^30^. As supplements to SOC, various immunotherapies have been explored in glioblastoma^31^-^34^, primarily based on the success in peripheral cancers. Among them, tumor vaccines can potentially serve as complementary monotherapy or boost the clinical efficacy with other immunotherapies. Four generic types of glioblastoma vaccines have been explored: peptide, dendritic cell (DC), mRNA, and viral vector vaccines. Previous studies have suggested that single-peptide therapeutic vaccination had limited efficacy in tumor control. To boost effectiveness, a much more effective immune recognition of cancer cells needs to be jumpstarted. The general elements for effective vaccine design include a tumor antigen, a vehicle to transport the antigen to its target, and an adjuvant or **“**danger signal”. The synergistic integration of these elements ultimately determines the potency and spectrum of the resulting anti-tumor immune response.

Here we used an artificial antigen, ovalbumin, to compare the therapeutic efficacy of different delivery strategies in a well-established immunologic model system^23^. Natively, glioblastoma has low a tumor mutational burden (TMB) and few neoantigens^5^, ^35^, ^36^. Currently, these neoantigens are mainly selected based on single nucleotide mutations, but additional neoantigens originating from structural variation, frameshifting, gene fusion, and cancer-associated chromosomal abnormalities are being explored. Given the current limitations on antigen choices, improving the delivery platform and adjuvant has become all the more important in designing more effective glioblastoma vaccines. Future experimental work in the CT2a model could target mPomgnt1_R497L_, mEpb4_H471L_, and mPlin2_G332R_ 34, 37.

Transport vehicle. While many different mRNA delivery vehicles have been suggested based on material design principles, we first looked at the unique physiological and cellular challenges in glioblastoma. In particular, we aimed to target the central nervous system APC capable of priming antitumor CD8 T-cell responses, a cell population that has recently been identified^36^. The transport vehicle used here, based on crosslinked bis-succinyl β-cyclodextrin, showed high accumulation in glioblastoma APC, while cellular uptake in these cells is likely mediated via carbohydrate moieties. A further unique feature of RNC is their surface fluorination with ferrocenyl polyfluorocarbon, which results in efficient lysosomal escape and significantly higher mRNA expression compared to LNP-mediated mRNA delivery. This effect is presumably attributed to the enhanced ability of fluorinated RNC to cross cellular lipid bilayers^14^. Fluorination has also been used to enhance the intracellular delivery efficiency of LNP^38^, polymers^39^, ^40^, proteins, and small molecules^41^. In the circulation studies, the blood half-life experiment was designed to characterize the biphasic pharmacokinetics of iRNCs rather than to benchmark against commercial LNPs. Biodistribution analysis further showed preferential accumulation in glioblastoma (4.7 × 10⁷ ± 1.2 × 10⁷ ROI) relative to spleen (2.2 × 10⁷ ± 0.1 × 10⁷) and lymph nodes (1.8 × 10⁷ ± 0.09 × 10⁷), indicating that tumor-resident APCs receive the dominant nanoparticle exposure. This distribution pattern guided our focus on transfection analyses within the tumor microenvironment, while the low nanoparticle burden in distal lymphoid organs limited quantitative assessment of transfection in those tissues. Real-time intravital imaging in orthotopic glioma-bearing IL-12 reporter mice clearly demonstrated rapid vascular access, transvascular distribution, and functional engagement of tumor-resident myeloid cells by systemically administered iRNCs. We did not include a commercial LNP control, as there are fundamental differences in physiological behavior across the hundreds of published LNP studies 7, 42. In summary, the iRNC vehicle is extremely efficient because of its high tumoral APC targeting, efficient lysosomal escape, and high mRNA-protein expression.

Vaccine adjuvants for enhanced immuno-stimulation. Immunostimulatory components (adjuvants) are essential in enhancing the durability and magnitude of the immune response given the often low immunogenicity of cancer vaccines. The primary role of the adjuvant is to activate the innate immune system by introducing damage-associated molecular patterns (DAMPs) or pathogen-associated molecular patterns (PAMPs), induce cytokine production (IL12), and recruiting other immune cells to enhance the adaptive immune response. Based on the prior exploration of this field, we used the polymeric β-cyclodextrin (β-CD) platform as a supramolecular host^43^, ^44^ to deliver NFkB stimulating small molecules. Prior work had identified TLR7/8 agonists (e.g. R848) and cIAPi inhibitors (e.g. LCL-161) as powerful combinations to simultaneously activate the canonical and non-canonical NFkB pathways in APC^11^, ^45^-^47^. Alternative adjuvants are possible, such as poly I:C^48^, ^49^, among others. These, however, pose additional drug formulation challenges such as size and charge profile. Here we show highly effective immune cell stimulation in the glioblastoma TME as evidenced by the temporal IL12 induction kinetics. This, in turn, led to effective tumor control. The modular form of vaccination in RNC is unique because in a first dose the adjuvants could be loaded to initiate the T cell response. Upon re-dosing it may not be necessary to keep providing adjuvants, since one would be re-stimulating memory T cells. Conversely, LNP based vaccines are naturally adjuvanted, providing the adjuvant each time on re-dosing, i.e. inherently more toxic. In parallel, cyclodextrin nanoconstructs inducing IL12 have previously been tested for toxicity, including weight loss, blood, and histopathological analyses^11^, ^13^, ^50^. At therapeutic doses used, we did not observe major toxicities.

In summary, we demonstrate that the RNC734 platform functions as a potent systemic mRNA vaccine capable of eradicating glioblastoma in murine models. Moving forward, future efforts will focus on expanding this strategy to include native glioblastoma neoantigens, multi-antigen formulations, and rational combinations with complementary therapies, including immune checkpoint blockade and other immunomodulatory approaches.

## Materials and Methods

### Materials

All reagents and solvents were purchased from Thermo Fischer or Sigma-Aldrich and used as received. R848 (purity: 99.77%) and LCL161 (purity: 99.91%) were purchased from MedChemExpress and used as is. MilliQ water obtained from Waters filtration system. Ovalbumin (OVA) mRNA (m1Ψ) was purchased from Genscript. The OVA 323-339 I-Ad-restricted OVA MHC class II epitope and OVA 257-264 H-2Kb-restricted OVA MHC class I epitope were purchased from Invivogen.

### Synthesis

RNC synthesis. Bis succinyl beta cyclodextrin (b-s-CD) was synthesized in-house with an average of 2 succinyl groups per CD according to previously established protocols^11^, ^51^. For the core unit preparation, b-s-CD (DS 2.5, 10 mg, 6.99 μmol) was first activated with EDC (40.70 mg, 262.2 μmol, 15 eq.) and NHS (15.09 mg, 131.10 μmol, 7.5 eq.) in 240 µl of MES-Buffer (50 mM, pH=6.13). The quantity of linker was calculated according to the number of available activated carboxyl group. N1-(2-(4-(2-Aminoethyl)piperazin-1-yl)ethyl)ethane-1,2-diamine (NAPED) (1.88 mg, 8.74 μmol, 0.5 eq.) was dissolved in 60 µl MES-Buffer and introduced dropwise into the activated b-s-CD solution, after which the mixture was stirred for 24 h. The resulting particles were purified via filtration (30k MWCO cut-off filter) and then stored at -20 °C.

Ferrocenyl-perfluorooctylamine. To synthesize the product, ferrocenecarboxylic acid (100 mg, 0.43 mmol, 1.0 eq.) was dissolved in 4 mL of anhydrous dimethylformamide (DMF) along with PyBOP (400 mg, 0.77 mmol, 1.8 eq.) and 200 μL of N,N-diisopropylethylamine. In parallel, 1H,1H-perfluorooctylamine (640 mg, 1.6 mmol, 4.4 eq.) was separately dissolved in 2 mL of anhydrous DMF and then gradually introduced into the reaction mixture. The reaction proceeded at room temperature, with progress monitored using LC-MS. Upon completion, the solvent was removed via rotary evaporation, and residual solvents were eliminated by co-evaporation with toluene (three times) to obtain a dry solid. Purification of the crude product was conducted using normal-phase column chromatography (Sorbtech, spherical silica, 70Å, 20-45 μm) on a Biotage Isolera flash system, employing a linear gradient of ethyl acetate in hexanes (0% to 20%). The final product was characterized through LC-MS and NMR spectroscopy and had previously been characterized^14^.

Preparation of RNC complex for *in vitro* and *in vivo* applications. To prepare the RNC734 complex, 5 mg of cyclodextrin particles were dissolved in 90 μL of 1× phosphate-buffered saline (PBS, pH 7.4) and mixed to ensure complete dispersion. Separately, ferrocenyl-perfluorooctylamine (0.05 mg), R848 (0.2 mg), and LCL161 (0.3 mg) were dissolved in 5 μL of dimethyl sulfoxide (DMSO) and vortexed thoroughly to achieve homogeneity. The prepared DMSO solution was then added to the PBS suspension containing the cyclodextrin particles, followed by vigorous pipetting to facilitate efficient loading of the components onto the particles. For *in vivo* administration, 10 μg of mRNA was condensed into the preformed complex and injected into the tail vein of experimental models. For *in vitro* applications, 500 ng of mRNA was introduced to a culture of 10⁴ cells, while the RNC complex was added at a final NP ratio of 16, ensuring the presence of all functional components.

LNP. LNPs were prepared using SM-102 as the ionizable lipid, along with DSPC, cholesterol, and PEG-lipid at a molar ratio of 50:10:38.5:1.5^1^. Lipids were dissolved in ethanol and mixed with an acidified mRNA solution (25 mM sodium acetate buffer, pH 5.0 or 50 mM sodium citrate buffer, pH 4.0) at a 3:1 aqueous:ethanol ratio using a microfluidic mixer. The N:P ratio was set at 5.67. Formulations were dialyzed in PBS buffer (pH 7.1) or 20 mM Tris buffer (pH 7.3) with 8% of sucrose for 18 h, concentrated if needed, sterile-filtered (0.22 μm), and stored in PBS at 4 °C or Tris-sucrose buffer at -20 °C. DLS analysis confirmed particle sizes of 50-142 nm, with >69% RNA encapsulation and <3 EU/mL endotoxin. Lead formulations (66-107 nm) had >90% encapsulation.

Lipofectamine. A transfection complex was prepared by diluting 500 ng of mRNA in 25 μL of Opti-MEM (Gibco) and separately diluting Lipofectamine 2000 in 25 μL of Opti-MEM according to the manufacturer**'**s protocol. After a 5 min incubation at room temperature, the mRNA and Lipofectamine solutions were mixed and incubated for an additional 10 min to allow complex formation.

### Characterization

Particle size and surface charge. Particle size and surface charge for all nanoparticle formulations were characterized using dynamic light scattering (DLS) and zeta potential analysis, respectively, performed on a Malvern Zetasizer APS. DLS measurements were carried out using samples prepared at 5 mg mL-1 in PBS (0.5x), wherease zeta potential was assessed at 2 mg mL-1 in PBS (0.1x). All analyses were performed in DTS1170 cuvettes (Malvern) at 25 ºC. Absorbance and fluorescence spectra were obtained using a multimode microplate reader (Tecan Spark 500) in 96-well black polystyrene plates with transparent bottoms (Corning). DLS and zeta potential measurements were repeated for each scaled-up nanoparticle batch and verified prior to initiating both *in vitro* and *in vivo* studies.

Loading efficiency assay. RNC stock solutions (2.5 mg/mL in 0.5× PBS, pH 7.4) were prepared as the nanoparticle matrix. Small molecule adjuvants were dissolved in DMSO to generate concentrated stocks. The loading process involved mixing the RNC particles with drug solutions at final concentrations ranging from 0.1 to 52 mM in a buffer containing 10% DMSO. Loading efficiency was evaluated by monitoring the decrease in UV-visible absorbance (400-700 nm) relative to a no-payload control. Absorbance loss was interpreted as successful payload encapsulation. All assays were conducted in triplicate (N = 3).

Small molecule adjuvant release kinetics. The release profile of small molecules from RNC was measured using a dialysis-based method with a 3 kDa MWCO membrane (Pur-A-Lyzer Midi Dialysis Kit). Nanoparticles (200 mg) loaded with R848 (2.8 μM) and LCL-161 (15 μM) in 1 mL PBS (10% DMSO) were dialyzed against 5 mL of PBS at 37 °C with continuous stirring at 600 rpm. At various time points (0 to 10 h), 100 μL of external buffer was collected and analyzed via liquid chromatography-mass spectrometry (LC-MS). Signature retention times and m/z values were used to identify each compound (e.g., R848: 0.78 min, m/z 315; LCL-161: 1.01 min, m/z 501). Drug release percentages were calculated based on the area under the chromatographic peak compared to undialyzed controls. All measurements were performed in triplicate.

Nucleic acid release kinetics. The release behavior of nucleic acids was assessed using dialysis with a 1000 kDa MWCO membrane. RNC700 nanoparticles (1.6 mg) loaded with 100 μg of mRNA was suspended in 1 mL PBS and dialyzed against 5 mL of PBS at 37 °C under stirring (600 rpm). At designated intervals (0 to 6 h), 100 μL aliquots were withdrawn to measure released nucleic acid levels using the Ribogreen RNA quantification kit, following the manufacturer's instructions. Fluorescence was recorded (λ_ex = 485 nm; λ_em = 515 nm) after 5 min of room temperature incubation. Nucleic acid concentrations were interpolated from standard curves. All experiments were performed in triplicate.

Electron microscopy. Freshly prepared RNC734 (5 mg/mL in 1× PBS) was diluted with ultrapure water to achieve a final working concentration of 1 mg/mL. A small volume of the particle suspension was applied onto a TEM grid and allowed to adsorb for 1 min. The sample was subsequently subjected to negative stained with a 2% uranyl acetate solution for 15 min, and rinsed three times with ultrapure water. TEM imaging was performed using a JEOL 2100 instrument.

For cryo-EM grid preparation, RNC samples (0.1 mg mL^-1^) were loaded onto plasma glow-discharged 300-mesh gold lacey carbon grids with an ultrathin carbon coating (Ted Pella) and vitrified using Vitrobot (FEI) operated at 4 °C and 100% humidity. Blotted grids (blot time 4 sec and blot force for 5 sec) were injected into liquid ethane and changed to liquid nitrogen for storage. Prior to data acquisition, grids were first evaluated on a Talos Arctica microscope (ThermoFisher) operating at 200 keV and equipped with Gatan K3 direct electron detector at the Harvard Cryo-EM Center for Structural Biology to confirm appropriate ice thickness and particle distribution. Datasets were acquired on a Titan Krios microscope (ThermoFisher) operated at 300 keV and equipped with state-of-the-art electron detection capabilities such as BioQuantum K3 Imaging Filter (Gatan, slit width 20 eV). Data were acquired in super-resolution mode at a nominal magnification of 105,000x (0.415 Å per pixel) with defocus values ranging from -1.0 to -2.0 μm. Each image stack contained 60 frames, corresponding to a cumulative electron dose of 60.0 e^-^/Å^2^. Automated data acquisition was performed using SerialEM version 3.8.

### Cell models

Bone marrow-derived cells. Bone marrow-derived cells were isolated from wild-type mice (**Table 3**). To harvest whole bone marrow, femurs were collected and flushed with sterile PBS using a syringe fitted with a 28-gauge needle. RBC Lysis Buffer (BioLegend) was used to lyse red blood cells according to the manufacturer**'**s instructions. After collection, the cells were enumerated using Neubauer chamber and plated at 1.25 x 10^5^ cells per well into black (Ibidi, glass bottom for imaging) or transparent (NEST, for flow cytometry analysis) 96 well plates. Bone marrow-derived dendritic cells (BMDCs) were generated by supplementing the culture medium with recombinant murine GM-CSF (20 ng mL-^1^, BioLegend) and FLT-3L (200 ng mL^-1^) for a 7-day differentiation period, with media changes performed every 3-4 days.

Immortalized cell lines. Immortalized murine bone marrow-derived macrophages (iMACs), kindly provided by Charles L. Evavold (Ragon Institute, Harvard University), were employed for gene transfection and cytotoxicity studies. The cells were maintained in Dulbecco's Modified Eagle Medium (DMEM, Corning) containing 10% fetal bovine serum and 1% penicillin-streptomycin (Corning) at 37 °C in a 5% CO_2_ atmosphere. CT2A-OVA were obtained from Gavin Dunn (MGH) and used to assess the *in vivo* vaccination effect. Cells were maintained in Iscove's Modified DMEM (Corning). Once cultures reached confluency, they were passaged using 0.05% trypsin / 0.53 mM EDTA (Corning), and all *in vitro* experiments were conducted when cultures approached 90% confluency. Before use in cell-based assays, all RNC formulations were sterilized by filtration through a 0.22 μm membrane (VWR).

### Cellular uptake and toxicity

iMACs were seeded in 96 well plates at 1.5 × 10⁴ cells per well and allowed to adhere to bottom for 24 h at 37 °C with 5% CO_2_. RNC734 stock solution was prepared and serially diluted in culture medium to final concentrations ranging from 0.153 μg/mL to 10 mg/mL, maintaining a DMSO content of 0.5%. For all uptake and cytotoxicity experiments, each well received RNC734 corresponding to 500 ng of mRNA along with 0.4 μM R848 and 0.5 μM LCL161. For comparison, LNP-mRNA and lipofectamine-mRNA complexes were also prepared to deliver 500 ng of mRNA per well, without additional adjuvants. Cells were incubated with the formulations for 24 h. Cellular uptake was visualized by Hoechst 33342 staining for nuclei and AF647 fluorescence for labeled nanoparticles. Images were acquired using a fluorescence microscope, and nanoparticle uptake was quantified using ImageJ software based on fluorescence intensity. For cytotoxicity analysis, the medium was replaced with FluoroBrite DMEM containing 10% Alamar Blue reagent (Invitrogen), and cells were incubated for 1 h at 37 °C with 5% CO₂. Fluorescence was measured at λ_ex = 550 nm and λ_em = 590 nm. All conditions were tested in triplicate, and IC_50_ values were calculated based on normalized fluorescence intensity.

### Immunocytofluorescence imaging

iMACs were seeded in an 8-well Nunc™ Lab-Tek™ II Chamber Slide™ System (Thermo Scientific) at a density of 4 × 10⁴ cells per well and incubated at 37 °C with 5% CO₂ for 24 h to allow adherence. Cells were then treated with RNC734-AF647 for an additional 18 h. Following incubation, cells were rinced three times and stained with LumiTracker® Lyso Green (Lumiprobe, Ex: 450 nm / Em: 508 nm) for 10 min at 37 °C to label lysosomes. Cells were then blocked with 1% BSA solution for 30 min at room temperature to reduce non-specific binding. For antigen presentation analysis, cells were incubated with OVA257-264 (SIINFEKL) peptide bound to H-2Kb Monoclonal Antibody (1:100 dilution, Ex: 488 nm / Em: 520 nm) for 1 h at room temperature. After washing, cells were stained with Goat anti-Mouse IgG1 Cross-Adsorbed Secondary Antibody, PE (1:200 dilution, Ex: 496 nm / Em: 578 nm) at room temperature in the dark for 45 min. Finally, nuclei were counterstained with Hoechst 33342 (1 μM, Ex: 350 nm / Em: 461 nm) for 10 min before imaging. Fluorescence images were acquired using a fluorescence microscope with appropriate filter sets for each fluorophore.

### Histology

Frozen tissue sections were cut to 5 µm thickness, stored at -20 °C and then processed for immunofluorescence. Frozen tissue sections were thawed at room temperature, rehydrated with PBS, and blocked with Intercept Blocking buffer (LI-COR) for 30 min before antibody staining. Tissue sections were incubated with antibodies (**Table S2**) for 1-2 h and washed with PBS for 10 min three times. For secondary staining, samples were stained with secondary antibodies or streptavidin for 30 min, followed by PBS wash. An Olympus BX-63 microscope was used for image acquisition (Metamorph software version 7.10.4). CellProfiler version 4.1.8 was used for the quantification of immune cell infiltration.

### Flow cytometry

Antigen presentation by DCs. BMDCs were pretreated with each RNC formulation or PBS for 48 h. The expressed SIINFEKL on the surface of DCs was observed by APC-conjugated anti-MHC-I-SIINFEKL via flow cytometry. Cells were also stained with fluorophore-conjugated anti-MHC-II, anti-CD11c, anti-CD80, anti-CD86, anti-CCR7 and anti-CD40 for the analysis of DC activation status.

T-cell activation and proliferation. T cells were collected from the spleens of OT-I transgenic mice and isolated according to the manufacturer**'**s guidelines (EasySep Mouse T-cell Isolation Kit, STEMCELL Technologies). Prior to co-culture, BMDCs were stimulated with each RNC formulations, 1x cell stimulation cocktail for P.C, and 1x protein transport inhibitor cocktail for N.C for 48 h. Stimulated BMDCs (3.3 x 10^5^ cells) were then co-cultured with isolated T cells (4 x 10^5^ cells) and incubated for 48 h to facilitate T-cell proliferation and activation. T-cell proliferation was monitored using the Invitrogen CellTrace CFSE kit to track distinct generations of proliferating cells by dye dilution in the flow cytometry.

Surface marker staining. Single-cell suspensions from tumors or spleen were first blocked with TruStain anti-mouse CD16/32 (BioLegend, 156603) and incubated with antibodies against surface markers at 4 °C for 30 min, followed by live-dead staining (Fixable Aqua); cells were washed twice and resuspended in PBS before cytometry analysis. The following antibodies used in flow cytometry (FCM) were purchased from MBL bio: H-2Kb OVA G4 Tetramer-SIINFEKL (APC, TB-5001-2) and BioLegend and Invitrogen: anti-mouse CD3 (PE-Cy5, 100309), anti-mouse CD8a (FITC, MA5-16759), anti-mouse CD4 (BV785, 100552), anti-mouse CD11c (PE-eFluor610, 61-0114-82), anti-mouse MHC-II (AF700, 107622), anti-mouse CD80 (BV421, 104726), anti-mouse CD86 (BV785, 105043; FITC, 105005), anti-mouse F4/80 (PE-Cy7, 123114), anti-mouse CCR7 (PE, 120105), anti-mouse CD40 (PerCP-Cy5.5, 124626), and anti-mouse H-2Kb SIINFEKL (APC, 141606).

### IFNγ ELISpot

Following the isolation and processing of splenocytes or PBMDCs as described earlier, cells were seeded into 96-well round-bottom plates at a volume of 200 μL per well, ensuring each well contained cells from an individual mouse. To stimulate antigen-specific responses, wells were treated with 2 μg/mL of the relevant peptide. For CD8⁺ T-cell activation, the MHC class I-restricted OVA peptide epitope (OVA₂₅₇₋₂₆₄, SIINFEKLC; Invivogen) was used. Meanwhile, the MHC class II-restricted OVA peptide epitope (OVA₃₂₃₋₃₃₉, ISQAVHAAHAEINEAGR; Invivogen) was applied to assess CD4⁺ T-cell responses. The cells were incubated at 37°C for 48 h to allow for stimulation. After incubation, cells were harvested by centrifugation (1,200 rpm, 5 min, 4°C) and resuspended in 100 μL of sterile splenocyte media. Subsequently, the cells were incubated for an additional 36 h at 37°C before processing the ELISpot plates according to the manufacturer's instructions (Mabtech).

### Mouse models

All animals were bred and housed under specific pathogen free conditions at the Massachusetts General Hospital. Experiments were approved by the MGH Institutional Animal Care and Use Committee (IACUC) and were performed in accordance with MGH IACUC regulations. A total of n = 42 mice were used (**Table 3**). This included n = 35 for C57BL/6J, n = 3 for B6.129-IL12btm1Lky/J, n=3 for ROSAnT-nG, and n=1 for C57BL/6-Tg(TcraTcrb)1100Mjb/J.

Tumor cell implantation. For intracranial tumor implantations, 1×10^5^ CT2A-OVA cells were diluted in 2 μL of sterile PBS (Sigma-Aldrich) and precisely stereotactically implanted into the right basal ganglia of 10-12-week-old IL12-eYFP (C57Bl/6) mice. The implantation coordinates were set at 2 mm right lateral of the bregma and 0.5 mm anterior to the coronal suture, with an injection depth of 1 mm below the dural surface. Implantation was performed using a 10 μL Hamilton micro-syringe driven by a fine step stereotactic device (Kopf). For subcutaneous tumor implantations, 1×10^6^ CT2A-OVA cells were suspended in 100 μL of sterile PBS and injected into the flank of the mice via subcutaneous administration.

MR Imaging. MRI scans were conducted using a 4.7-T MR imaging unit (Bruker Pharmascan) on the designated animal at day 18 post-tumor inoculation. Imaging was performed under respiration-monitored isoflurane anesthesia to ensure stable physiological conditions. Coronal imaging sequences included pre- and post-contrast enhanced T1-weighted imaging. The imaging parameters were optimized for tissue contrast and resolution: repetition time (TR) of 700 ms, echo time (TE) of 14 ms, matrix size of 256 × 256, and slice thickness of 0.5 mm. A total of 12 sections were acquired for comprehensive coverage. Additionally, pre-enhanced T2-weighted imaging was performed to assess tissue characteristics. Imaging parameters for T2-weighted sequences included a TR of 4000 ms, TE of 53.3 ms, matrix size of 256 × 256, and slice thickness of 0.5 mm. Similar to T1-weighted imaging, 12 sections were acquired to ensure thorough evaluation. Tumor volumes were quantified using Horos image-processing software, facilitating ROI-based 3D analysis of Gd-DTPA enhanced T1-weighted MR images.

### Intravital imaging

Cranial windows implantation was performed following established protocols 52. The head of 10-12-week-old mice was shaved, animals were immobilized in a stereotactic frame (Kopf, Tujunga, CA), and the skull was sterilized with two cycles of betadine-isopropanol. A large oval skin area was removed, from behind the ears to between the eyes, surrounding the lambda and bregma sutures. The periosteum was pushed to the side, and all tissue on top of the skull was scraped off. The rim of the 5 mm circular section of the skull, excluding the lambda and bregma, was sanded down using a Dremel with a burr (Ideal Mircro-Drill) and removed to provide an opening to the brain. Using stereotaxic positioning, 2 µl of Optimum (Thermo Fisher Scientific) with a 1:1 mix of 10^5^ CT2A-OVA and CT2A-H2B-apple cells were injected at about 1 mm depth near the middle of the opening, avoiding vasculature. Gelfoam and saline were used to remove blood during surgery and after injection. A drop of saline and an 8 mm round cover glass were placed onto the opening. Super glue was used to attach only the rim of the cover glass to the skull, avoiding any contact between the adhesive and the brain. Then, dental cement was used to cement the cover glass onto the skull and to form an elevated rim for water immersion imaging.

Intravital confocla images were performed using a customized Olympus FV1000 microscope (Olympus America). Imaging utilized 2x (XLFluor, NA 0.14), 4x (UPlanSApo, NA 0.16), and 20x (XLUMPlanFL N, NA 1.0) water-immersion objectives. CT-2A H2B-apple tumor cells, RNC734-AF647, and vascular tracers were sequentially excited with 405, 473, 559, and 633 nm diode lasers, respectively, in combination with a DM-405/488/559/635 nm dichroic beam splitter. Emission signals were further separated using SDM-473, SDM-560, and SDM-640 beam splitters and collected through BA430-455, BA490-540, BA575-620, and BA655-755 emission filters (Olympus America). Laser power was minimized to prevent photobleaching, phototoxicity, and tissue injury. Image processing was carried out in Fiji (ImageJ2, Vers.2.3/1.54mf).

### Statistics

All statistical analyses were performed using GraphPad Prism 9 software, and results are expressed as mean ± standard deviation. Group comparisons were analyzed using two-tailed Student's t-tests. Differences with p-values below 0.05 were regarded as statistically significant, whereas those above 0.05 were interpreted as not significant (n.s.).

## Supplementary Material

Supplementary figures.

## Figures and Tables

**Table 1:** ** Overview of mRNA delivery constructs synthesized.** The ratio indicates the w/w% ratio of mRNA to RNC.

Type	Zeta potential(mv)	Size	PDI	mRNA	Condensor	Drug load	Comment
Naked mRNA	NA	1438 n.t.	NA	OVA	None	NA	
Lipofectamine	NA	110 ± 4.1 nm	0.226	OVA	DOSPA, DOPE	NA	
LNP		98 ± 9.3 nm	0.151	OVA	SM102	NA	
RNC730	13.1 ± 2.1	79.3 ± 2.1 nm	0.293	None	NAPED	None	Only ferrocenyl perfluorooctylamine loaded
RNC731	11.8 ± 1.1	194.2 ± 1.4 nm	0.312	OVA	NAPED	None	Ratio 16
RNC734^Lo^	12.3 ± 0.9	154 ± 6.5 nm	0.297	OVA	NAPED	R848, LCL161	Ratio 4
RNC734^Hi^	10.9 ± 1.5	200 ± 3.6 nm	0.133	OVA	NAPED	R848, LCL161	Ratio 16

**Table 2:** ** Antibodies used.** IF: immunofluorescence; FLOW: Flow cytometry.

Antibody	Application	Target population	Source	Dye	Catalog #	Clone
CD45	IF	Hematopoetic Cells	BioLegend	AF488	368535	2D1
CD4	IF	CD4 T Cells	BioLegend	AF488	100532	RM4-5
CD8-biotin	IF	CD8 T Cells	BioLegend	N/A	100704	53-6.7
FoxP3	IF	Regulatory T cells	BioLegend	AF647	126408	MF14
F4/80	IF	Macrophage	BioLegend	AF488	123119	BM8
CD11c-biotin	IF	DC	BioLegend	N/A	117303	N418
MHC-II	IF	Antigen-presenting cell	BioLegend	AF647	107617	M5/114.15.2
Ly-6C	IF	Monocyte	BioLegend	AF750	128003	HK1.4
TMEM119	IF	Microglia	Abcam	AF647	ab209064	28-3
Goat anti-rabbit secondary antibody	IF	Rabbit IgG	Abcam	AF647	Ab150083	Polyclonal
OVA257-264 (SIINFEKL) peptide bound to H-2Kb monoclonal antibody	IF	Myeloid cell	eBioscience		145743-82	eBio25-D1.16 (25-D1.16)
Goat anti-Mouse IgG1 cross-adsorbed secondary antibody	IF	Mouse IgG1	Invitrogen	PE	P-21129	ABCG2
Streptavidin	IF	Biotin-conjugated primary antibody	Thermo	AF750	S21384	N/A
CD45.1	Flow	Immune cells	BioLegend	BV650	103151	30-F11
CD8alpha	Flow	CD8 T Cells	Invitrogen	FITC	MA5-16759	KT15
CD11c	Flow	Antigen-presenting cell	Thermo	PE-eF610	61-0114-82	N418
F4/80	Flow	Macrophage	Thermo	PE-Cy7	123114	BM8
CD80	Flow	Co-stimulation	BioLegend	BV421	104726	16-10A1
MHC II	Flow	Antigen-presenting cell	Thermo	AF700	107622	M5/114.15.2
CD40	Flow	Co-stimulation	Biolegend	PerCP-Cy5.5	124626	3/23
Live/Dead Fixable Aqua	Flow	Live Dead Marker	Thermo		L34966	
CD86	Flow	Co-stimulation	Biolegend	BV785	105043	GL-1
CD86	Flow	Co-stimulation	Biolegend	FITC	105005	GL-1
CCR7	Flow	Chemokine Receptor	BioLegend	PE	120105	4B12
MHC Tetramer H-2kb bound to SIINFEKL	Flow	Ova-specific CD8 T cells	MBL	APC	TB-5001-2	
H-2kb bound to SIINFEKL	Flow	Ova Peptide Presented on MHC-I	BioLegend	APC	141606	25-D1.16
CD3epsilon	Flow	T cells	BioLegend	PE-Cy5	100309	145-2C11
CD4	Flow	CD4 T cells	BioLegend	BV785	100552	RM4-5

**Table 3:** Cell lines and mouse models

Model	Description	Source	Catalog	Number	Comment
C57BL/6J	Immunocompetent host	Jackson Laboratory	Stock# 000664 RRID:IMSR_JAX:000664	35	Wild-type
B6.129-IL12btm1Lky/J	IL12-eYFP producing myeloid cells	Jackson Laboratory	Stock# 006412 RRID:IMSR_JAX:006412	3	IL12 reporter
ROSAnT-nG	Dual-color fluorescent tracking of Cre recombinase activity	Jackson Laboratory	Stock# 023035 RRID:IM_SR_JAX:023035	3	Visualization of Cre-mediated recombination events.
C57BL/6-Tg(TcraTcrb)1100Mjb/J	Recognizes the OVA peptide SIINFEKL presented by H-2Kb	Jackson Laboratory	Stock# 003831 RRID:IM_SR_JAX:003831	1	CD8+ T cell responses to antigens
CT2A	Wild-type murine glioblastoma	Samuel Rabkin, MGB Xandra Breakefield, MGB	PMID: 1418222	NA	Efficacy studies
CT2A-H2Bapple	Histone transfected fluorescent CT2A	Katy Yang, MGB	NA	NA	Intravital imaging
CT2A-OVA	OVA transfected CT2A	Gavin Dunn, MGB	NA	NA	Efficacy studies
iMACs	Immortalized murine bone marrow-derived macrophages	Charles L. Evavold, MGB	PMID: 34289345	NA	Uptake and toxicity experiments

## References

[1] Hou X, Zaks T, Langer R, Dong Y (2021). Lipid nanoparticles for mRNA delivery. Nat Rev Mater.

[2] Han X, Alameh M-G, Butowska K, Knox JJ, Lundgreen K, Ghattas M (2023). Adjuvant lipidoid-substituted lipid nanoparticles augment the immunogenicity of SARS-CoV-2 mRNA vaccines. Nat Nanotechnol.

[3] Weiss AM, Hossainy S, Rowan SJ, Hubbell JA, Esser-Kahn AP (2022). Immunostimulatory Polymers as Adjuvants, Immunotherapies, and Delivery Systems. Macromolecules.

[4] Sharma P, Hoorn D, Aitha A, Breier D, Peer D (2024). The immunostimulatory nature of mRNA lipid nanoparticles. Adv Drug Deliv Rev.

[5] Mendez-Gomez HR, DeVries A, Castillo P, von Roemeling C, Qdaisat S, Stover BD (2024). RNA aggregates harness the danger response for potent cancer immunotherapy. Cell.

[6] Yin D, Zhong Y, Ling S, Lu S, Wang X, Jiang Z Dendritic-cell-targeting virus-like particles as potent mRNA vaccine carriers. Nat Biomed Eng. 2024.

[7] Das R, Halabi EA, Fredrich IR, Oh J, Peterson HM, Ge X (2023). Hybrid LNP Prime Dendritic Cells for Nucleotide Delivery. Adv Sci (Weinh).

[8] Krishnan N, Kubiatowicz LJ, Holay M, Zhou J, Fang RH, Zhang L (2022). Bacterial membrane vesicles for vaccine applications. Adv Drug Deliv Rev.

[9] Meng Z, Zhang Y, Zhou X, Ji J, Liu Z (2022). Nanovaccines with cell-derived components for cancer immunotherapy. Adv Drug Deliv Rev.

[10] Weissleder R, Nahrendorf M, Pittet MJ (2014). Imaging macrophages with nanoparticles. Nat Mater.

[11] Fredrich IR, Halabi EA, Kohler RH, Ge X, Garris CS, Weissleder R (2023). Highly Active Myeloid Therapy for Cancer. ACS Nano.

[12] Enbergs N, Halabi EA, Goubet AG, Schleyer K, Fredrich IR, Kohler RH (2024). Pharmacological Polarization of Tumor-Associated Macrophages Toward a CXCL9 Antitumor Phenotype. Adv Sci (Weinh).

[13] Kim HS, Simpson GG, Carrothers J, Nguyen YTM, Kohler R, Hong S (2025). A Myeloid Cell-Targeted Immunostimulant Cocktail (MyTai) Enhances Cancer Immunotherapy. ACS Nano.

[14] Kim HS, Simpson GG, Fei F, Garris C, Weissleder R (2025). Fluorinated Ribonucleocarbohydrate Nanoparticles Allow Ultraefficient mRNA Delivery and Protein Expression in Tumor-Associated Myeloid Cells. J Am Chem Soc.

[15] Rodell CB, Arlauckas SP, Cuccarese MF, Garris CS, Li R, Ahmed MS (2018). TLR7/8-agonist-loaded nanoparticles promote the polarization of tumour-associated macrophages to enhance cancer immunotherapy. Nat Biomed Eng.

[16] Stopforth RJ, Ward ES (2020). The Role of Antigen Presentation in Tumor-Associated Macrophages. Crit Rev Immunol.

[17] Kerdidani D, Aerakis E, Verrou K-M, Angelidis I, Douka K, Maniou M-A (2022). Lung tumor MHCII immunity depends on *in situ* antigen presentation by fibroblasts. J Exp Med.

[18] Zimmermannova O, Ferreira AG, Ascic E, Velasco Santiago M, Kurochkin I, Hansen M (2023). Restoring tumor immunogenicity with dendritic cell reprogramming. Sci Immunol.

[19] Oh J, Hoelzl J, Carlson JCT, Ruben B, Peterson HM, Faquin WC (2025). Spatial analysis identifies DC niches as predictors of pembrolizumab therapy in head and neck squamous cell cancer. Cell Rep Med.

[20] Jhunjhunwala S, Hammer C, Delamarre L (2021). Antigen presentation in cancer: insights into tumour immunogenicity and immune evasion. Nat Rev Cancer.

[21] Kim HY, Li R, Ng TSC, Courties G, Rodell CB, Prytyskach M (2018). Quantitative Imaging of Tumor-Associated Macrophages and Their Response to Therapy Using ^64^Cu-Labeled Macrin. ACS Nano.

[22] Kartal B, Garris CS, Kim HS, Kohler RH, Carrothers J, Halabi EA (2024). Targeted SPP1 Inhibition of Tumor-Associated Myeloid Cells Effectively Decreases Tumor Sizes. Adv Sci (Weinh).

[23] Kataoka K, Shiraishi Y, Takeda Y, Sakata S, Matsumoto M, Nagano S (2016). Aberrant PD-L1 expression through 3'-UTR disruption in multiple cancers. Nature.

[24] Baharom F, Ramirez-Valdez RA, Khalilnezhad A, Khalilnezhad S, Dillon M, Hermans D (2022). Systemic vaccination induces CD8^+^ T cells and remodels the tumor microenvironment. Cell.

[25] Rojas LA, Sethna Z, Soares KC, Olcese C, Pang N, Patterson E (2023). Personalized RNA neoantigen vaccines stimulate T cells in pancreatic cancer. Nature.

[26] Wu D, Chen Q, Chen X, Han F, Chen Z, Wang Y (2023). The blood-brain barrier: structure, regulation, and drug delivery. Signal Transduct Target Ther.

[27] Alieva M, Ritsma L, Giedt RJ, Weissleder R, van Rheenen J (2014). Imaging windows for long-term intravital imaging: General overview and technical insights. Intravital.

[28] Pittet MJ, Garris CS, Arlauckas SP, Weissleder R (2018). Recording the wild lives of immune cells. Sci Immunol.

[29] Miller MA, Weissleder R (2017). Imaging the pharmacology of nanomaterials by intravital microscopy: Toward understanding their biological behavior. Adv Drug Deliv Rev.

[30] Nam JY, de Groot JF (2017). Treatment of Glioblastoma. J Oncol Pract.

[31] Chiocca EA, Nassiri F, Wang J, Peruzzi P, Zadeh G (2019). Viral and other therapies for recurrent glioblastoma: is a 24-month durable response unusual. Neuro Oncol.

[32] Dyck L, Mills KHG (2017). Immune checkpoints and their inhibition in cancer and infectious diseases. Eur J Immunol.

[33] Kamiya-Matsuoka C, Gilbert MR (2015). Treating recurrent glioblastoma: an update. CNS Oncol.

[34] Dunn GP, Cloughesy TF, Maus MV, Prins RM, Reardon DA, Sonabend AM (2020). Emerging immunotherapies for malignant glioma: from immunogenomics to cell therapy. Neuro Oncol.

[35] Xiong Z, Raphael I, Olin M, Okada H, Li X, Kohanbash G (2024). Glioblastoma vaccines: past, present, and opportunities. EBioMedicine.

[36] Bowman-Kirigin JA, Desai R, Saunders BT, Wang AZ, Schaettler MO, Liu CJ (2023). The Conventional Dendritic Cell 1 Subset Primes CD8+ T Cells and Traffics Tumor Antigen to Drive Antitumor Immunity in the Brain. Cancer Immunol Res.

[37] Liu CJ, Schaettler M, Blaha DT, Bowman-Kirigin JA, Kobayashi DK, Livingstone AJ (2020). Treatment of an aggressive orthotopic murine glioblastoma model with combination checkpoint blockade and a multivalent neoantigen vaccine. Neuro Oncol.

[38] Zhang H, Meng C, Yi X, Han J, Wang J, Liu F (2024). Fluorinated Lipid Nanoparticles for Enhancing mRNA Delivery Efficiency. ACS Nano.

[39] Wang M, Liu H, Li L, Cheng Y (2014). A fluorinated dendrimer achieves excellent gene transfection efficacy at extremely low nitrogen to phosphorus ratios. Nat Commun.

[40] Xu J, Lv J, Zhuang Q, Yang Z, Cao Z, Xu L (2020). A general strategy towards personalized nanovaccines based on fluoropolymers for post-surgical cancer immunotherapy. Nat Nanotechnol.

[41] Li J, Wu Y, Xiang J, Wang H, Zhuang Q, Wei T (2023). Fluoroalkane modified cationic polymers for personalized mRNA cancer vaccines. Chem Eng J.

[42] Jiang AY, Witten J, Raji IO, Eweje F, MacIsaac C, Meng S (2024). Combinatorial development of nebulized mRNA delivery formulations for the lungs. Nat Nanotechnol.

[43] Hapiot F, Tilloy S, Monflier E (2006). Cyclodextrins as supramolecular hosts for organometallic complexes. Chem Rev.

[44] Davis ME, Brewster ME (2004). Cyclodextrin-based pharmaceutics: past, present and future. Nat Rev Drug Discov.

[45] Koch PD, Rodell CB, Kohler RH, Pittet MJ, Weissleder R (2020). Myeloid Cell-Targeted Nanocarriers Efficiently Inhibit Cellular Inhibitor of Apoptosis for Cancer Immunotherapy. Cell Chem Biol.

[46] Koch PD, Pittet MJ, Weissleder R (2020). The chemical biology of IL-12 production via the non-canonical NFkB pathway. RSC Chemical Biology.

[47] Lugani S, Halabi EA, Oh J, Kohler RH, Peterson HM, Breakefield XO (2023). Dual Immunostimulatory Pathway Agonism through a Synthetic Nanocarrier Triggers Robust Anti-Tumor Immunity in Murine Glioblastoma. Adv Mater.

[48] De Waele J, Verhezen T, van der Heijden S, Berneman ZN, Peeters M, Lardon F (2021). A systematic review on poly(I:C) and poly-ICLC in glioblastoma: adjuvants coordinating the unlocking of immunotherapy. J Exp Clin Cancer Res.

[49] Kim HS, Halabi EA, Enbergs N, Kohler RH, Fei F, Garris CS (2024). A non-lipid nucleic acid delivery vector with dendritic cell tropism and stimulation. Theranostics.

[50] Kaiser Y, Garris CS, Marinari E, Kim HS, Oh J, Pedard M Targeting immunosuppressive myeloid cells via implant-mediated slow release of small molecules to prevent glioblastoma recurrence. Nat Biomed Eng. 2025.

[51] García A, Leonardi D, Lamas MC (2016). Promising Applications in Drug Delivery Systems of a Novel β-Cyclodextrin Derivative Obtained by Green Synthesis. Bioorg Med Chem Lett.

[52] Mostany R, Portera-Cailliau C (2008). A craniotomy surgery procedure for chronic brain imaging. J Vis Exp.

