# Supplementary material for: A ribonucleotide carbohydrate system (iRNC) enhances antigen presentation and controls glioblastoma
Source: Theranostics. 2026 Jan 14;16(8):3912–27. doi: 10.7150/thno.127558 (PMC12905671; doi:10.7150/thno.127558)
Supplement: Supplementary file 1 — Supplementary figures. [file thnov16p3912s1.pdf]

Supplemental information

A ribonucleotide carbohydrate system (iRNC) enhances antigen presentation and controls glioblastoma

Hyung Shik Kim[1,4], Juhyun Oh[1], Jueun Jeon[1], Fan Fei[1], Sepideh Parvanian[1], Rainer Kohler[1], Christopher Garris[1,2], Ralph Weissleder[1,3]

[1] Center for Systems Biology, Massachusetts General Hospital, 185 Cambridge St, CPZN 5206, Boston, MA 02114,

[2] Department of Pathology, Massachusetts General Hospital, Boston, MA 02114

[3] Department of Systems Biology, Harvard Medical School, 200 Longwood Ave, Boston, MA 02115

[4] Graduate School for Biomedical Science & Engineering, Hanyang University, 04763, Seoul, Korea.

Corresponding author:
*R. Weissleder, MD, PhD
Center for Systems Biology
Massachusetts General Hospital 185 Cambridge St, CPZN 5206 Boston, MA, 02114
617-726-8226
rweissleder@mgh.harvard.edu

Figure. S1. A. EDS mapping of a single RNC particle (190 ±3.5 nm). **B.** Energy-dispersive X-ray spectra.

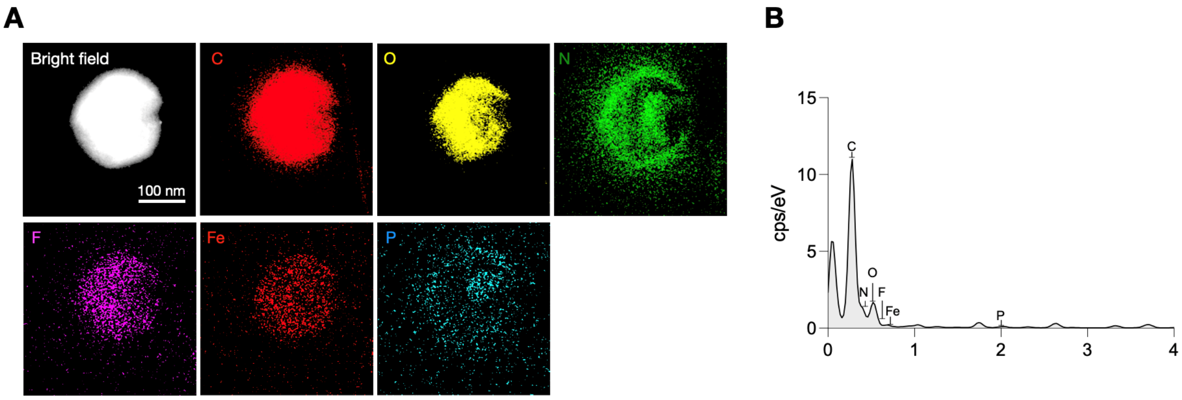

Figure. S2. Characterization of drug loading and release from iRNCs. A. Loading efficiency of small-molecule adjuvants assessed by turbidity-based absorbance measurements (N = 3; see Methods for details). For mRNA loading efficiency, refer to gel retardation analysis shown in **Figure. 2B. B.** Time-dependent release profiles of encapsulated mRNA and small molecules under physiological conditions (PBS, 37 °C) showing a characteristic sigmoidal pattern over 10 h (N = 3).

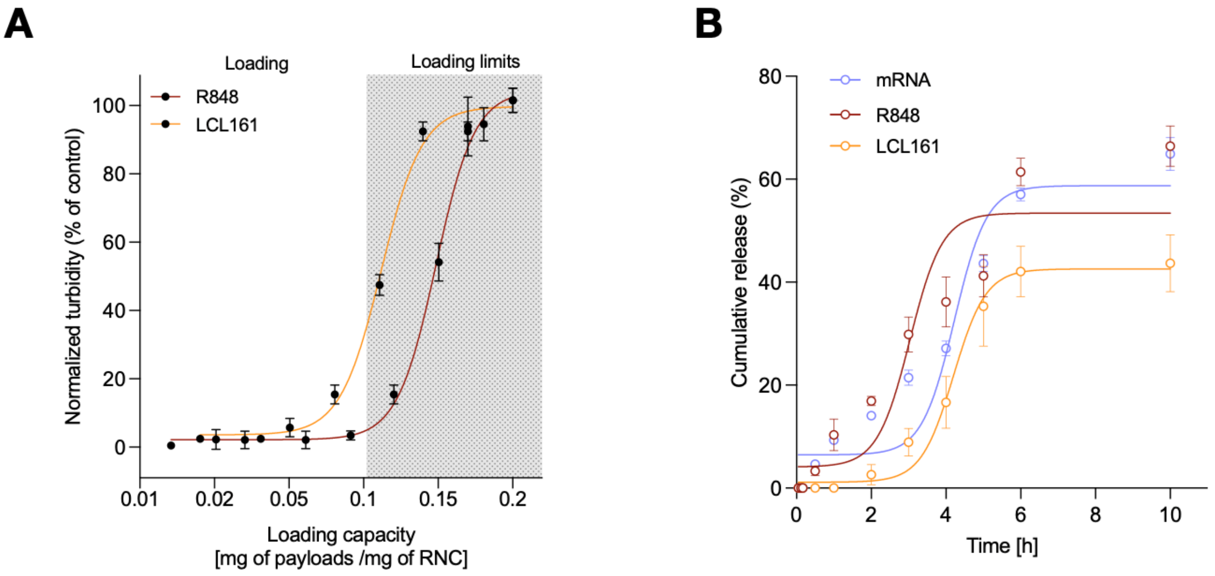

Figure. S3. Cellular uptake of RNC734 and cytotoxicity analysis. A. Fluorescence images of macrophages (IMACs) treated with RNC734-AF647 (yellow)at varying concentrations (0–10,000 μg/mL). **B.** Quantification of cellular uptake, demonstrating dose-dependent internalization. **C.** Cell viability assay indicating no significant toxicity up to 10,000 μg/mL.

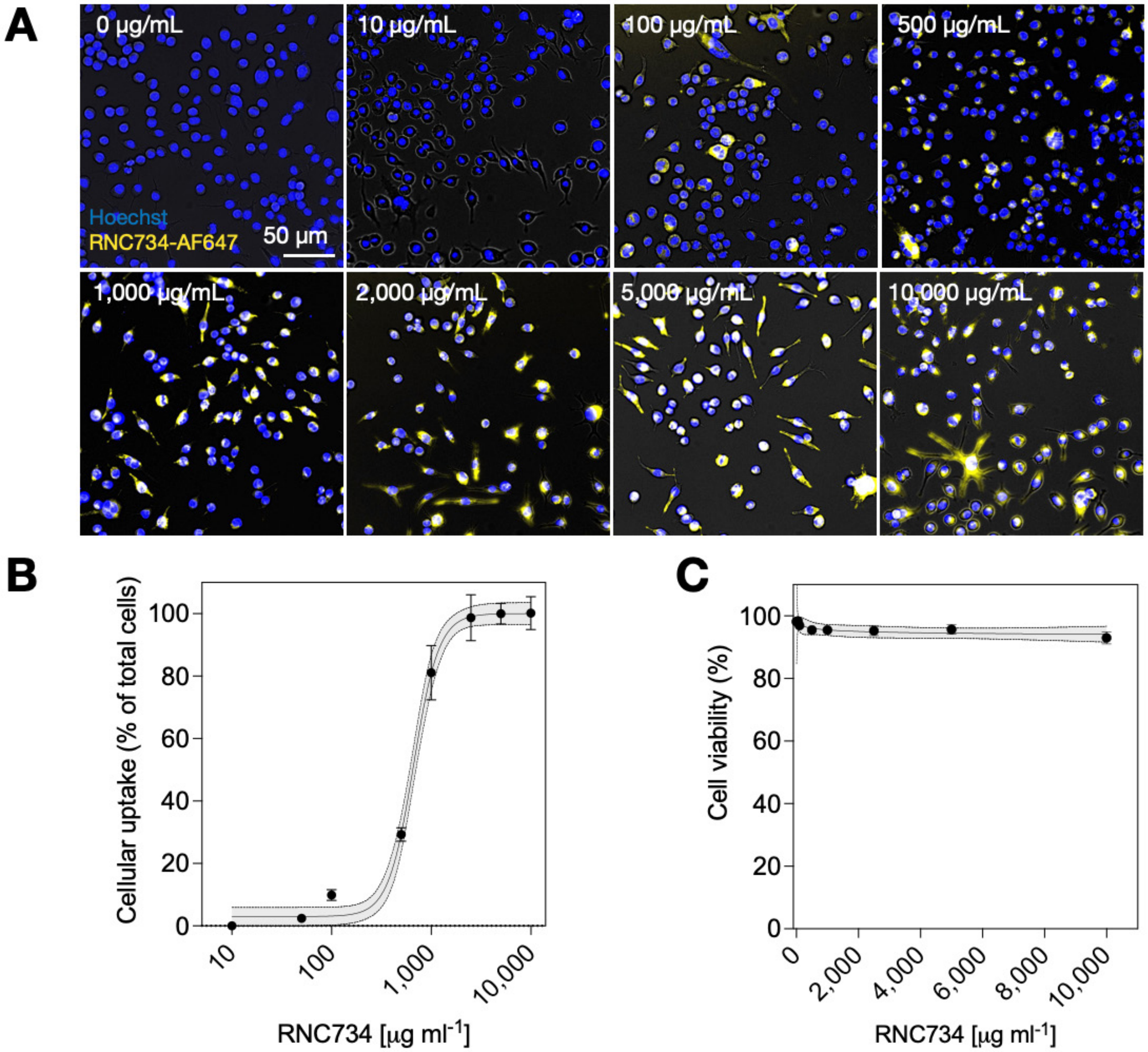

Figure. S4. Confocal Imaging of BMDC following RNC734 exposure
BMDC were obtained from BL donor mice and incubated with RNC734-AF647 (white) for 18 h. Cells were then washed and stained with H2kb SIINFEKL antibody (red), lysotracker (green), and Hoechst33342 (blue). **A.** Images show dendritic cell accumulation of RNC734 and efficient cell surface staining for OVA tetramer (SIINFEKL). **B.** Higher resolution images at 24 h post-treatment reveal RNC734 nanoparticles escaping from lysosomes, as indicated by reduced co-localization with Lysotracker staining.

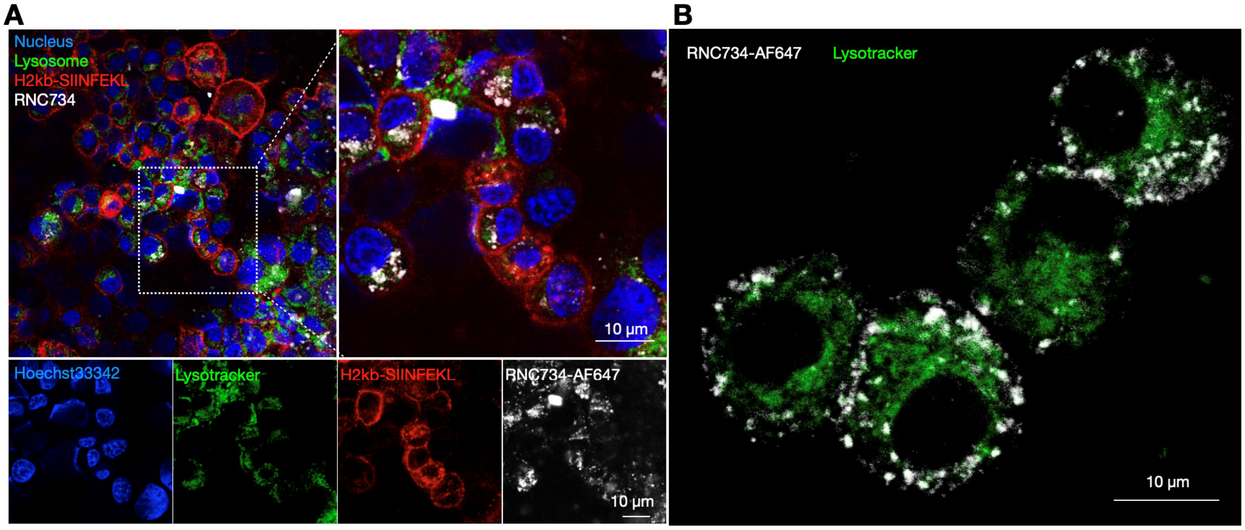

Figure. S5. Interferon-gamma (IFN-γ) production.
A. Splenocytes from OT-I transgenic donor mice were incubated with RNC pretreated BMDCs for 72 h, and their IFN production was determined by ELISpot. The circles correspond to wells with cells from 3 different experiments. The BMDC treatment groups include free OVA mRNA, RNC730, RNC731, or RNC734, while 1x cell stimulation cocktail (eBioscience) served as a positive control, and 1x protein transport inhibitor cocktail (eBioscience) treated cells as a negative control. **B.** Quantification of purple-stained spots from A. Note the highest IFN-γ production with RNC734. The compositions of RNC730, RNC731, and RNC734 are detailed in **Table 1**.

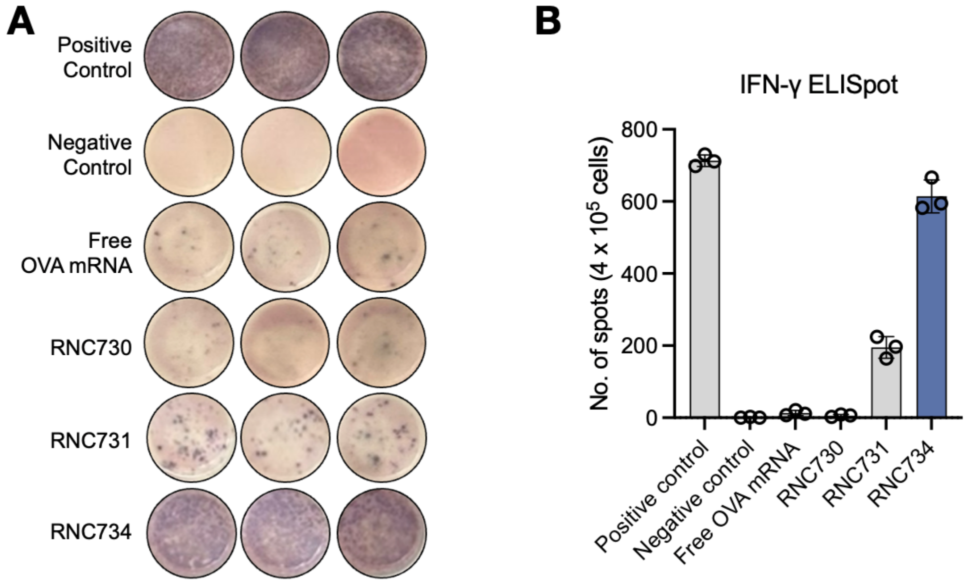

Figure. S6. CD8 T-cell proliferation assays following mRNA treatments
A. OT-1 CD8+ T cell expansion was measured after co-culture with pretreated BMDCs for 72 h using the carboxyfluorescein succinimidyl ester (CFSE) dilution method. The pretreated BMDCs were obtained from BL6 donor mice (n=1) and differentiated into DC by adding 20 ng mL-1 recombinant murine GM-CSF (BioLegend) and 200 ng mL-1 FLT-3L to cell culture media for 7 days. New media was added every 3-4 days. The BMDC were then exposed to either free OVA mRNA, RNC730, RNC731, or RNC734 for 24 h. The graphs show T-cell proliferation for the different treatment groups. **B.** Quantification of naïve OT-1 CD8+ T cell expansion. RNC734 showed the highest OT-1 CD8+ T proliferation. Each round datapoint represents one well. The compositions of RNC730, RNC731, and RNC734 are detailed in **Table 1**.

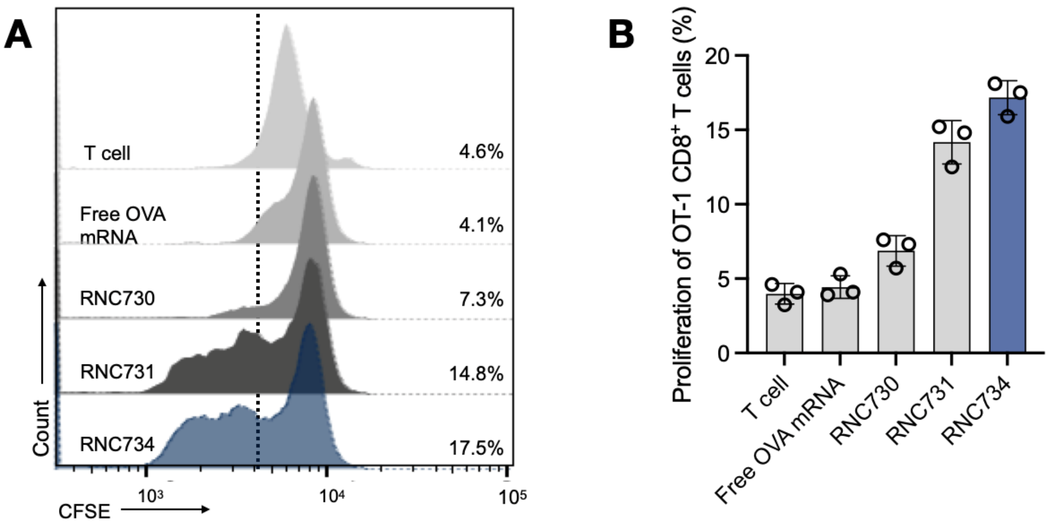

Figure. S7. Biodistribution of fluorescently labeled RNC in tumor-bearing mice
CT2a tumor-bearing mice were injected with 5 mg/kg of RNC734 labeled with i) Alexa Fluor 647 (RNC734-AF647) or ii) unlabeled (control). Organs were harvested after 24 h and imaged in well plates using an epifluorescence imaging system. **A**. Representative images for RNC734-AF647 injected mice demonstrates the material to accumulate in various organs, including CT2A tumor containing brain, Liver, lung, and kidneys. **B.** Note that the control injected mice (nonfluorescent RNC734) show no tissue fluorescence in the 650 nm channel.

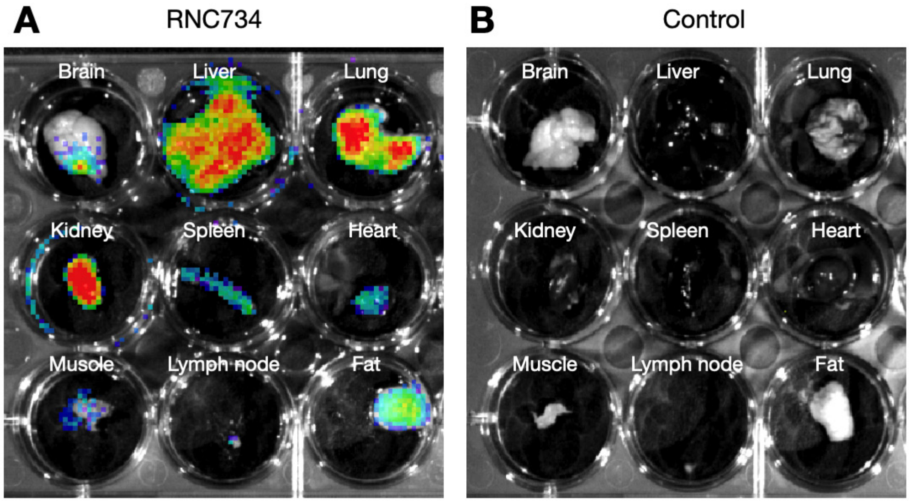

Figure. S8. Flow Cytometry Analysis of RNC Uptake in Tumor-Associated Immune Cells. **A.** Gating strategy. **B.** Heatmap of cellular uptake of RNC734 in the TME of three different tumor-bearing mice**.** RNC734 predominantly accumulates in tumor-associated macrophages (CD11b+, F4/80+) and dendritic cells (CD11c+, MHCII+) as opposed to other immune cell types in the TME.

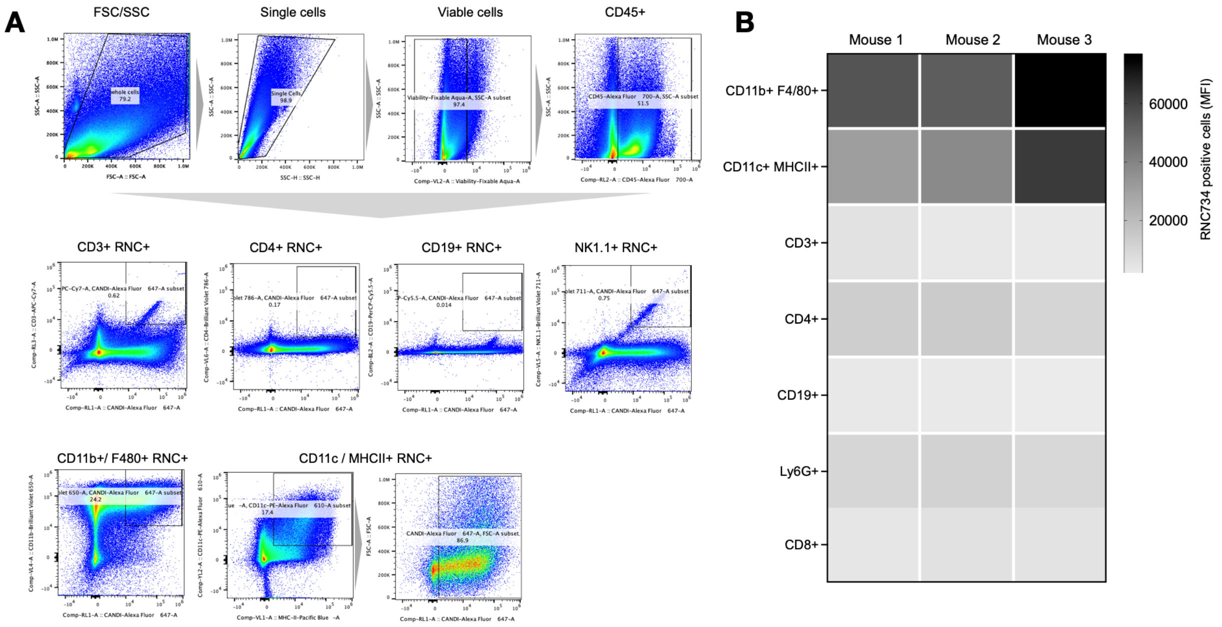

Figure. S9. Evaluation of Transfected Cell Types in Tumors Using the ROSAnT-nG Mouse Model.
A. Schematic overview of the ROSAnT-nG mouse model used to assess transfected cell types in tumors following systemic administration of RNC734 carrying Cre mRNA. Cre recombinase-mediated recombination results in a transition from tdTomato to eGFP expression, which is detected in transfected cells by flow cytometry. **B-C.** Flow cytometry analysis of eGFP-positive cells in tumor lysates, confirming transfection and recombination within the tumor microenvironment. **D.** Quantification of the proportion of eGFP+ cells within the tumor. Preferentially targeted cell types expressing eGFP include TAM (CD11b+ F4/80+) and DC (CD11c+ MHCII+).

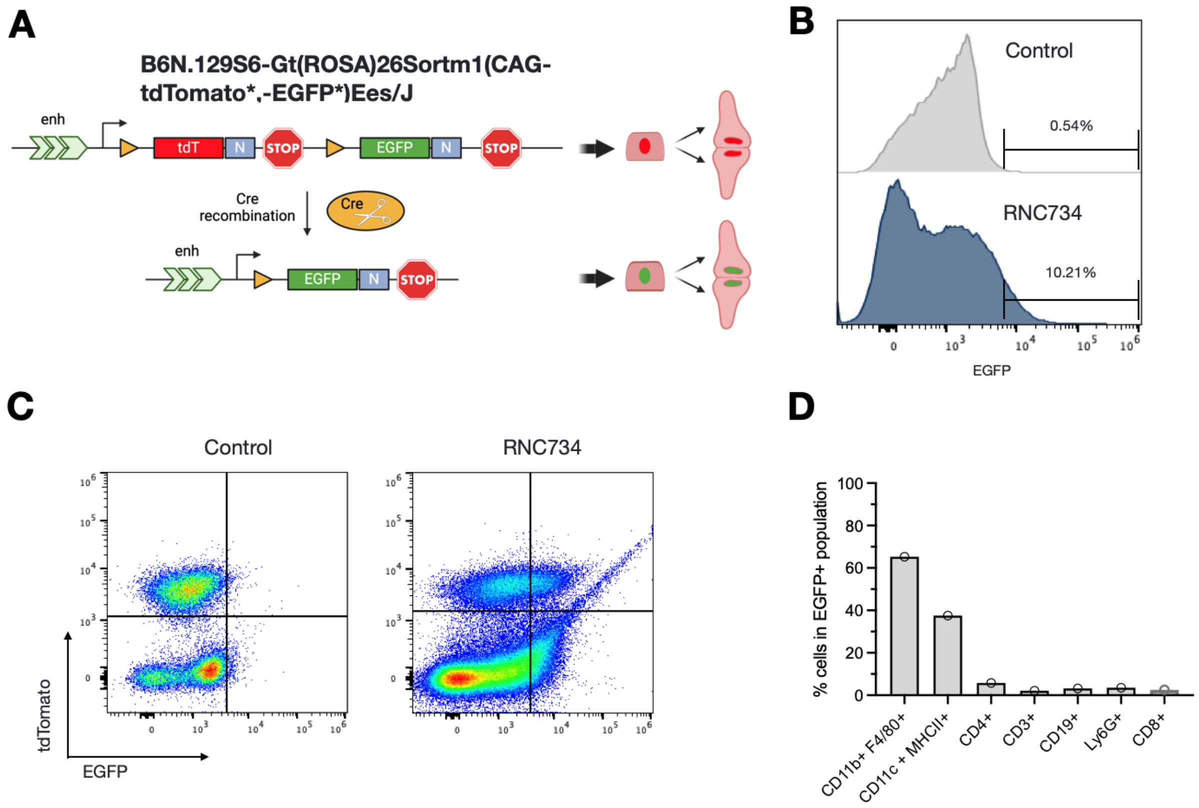

Figure. S10. Immunohistochemistry of CT2A tumors following RNC734 prophylactic trial. Mice with orthotopically implanted CT2A tumors were treated with systemic administration of RNC734 or PBS (control). On day 10 post-tumor inoculation (see **Figure. 5**), tumors were resected, fixed, and processed for immunohistochemistry. **A.** Representative panels from RNC734 treated mice show immune cell infiltration of glioblastoma. **B.** PBS injected mice showed immune deserted glioblastoma

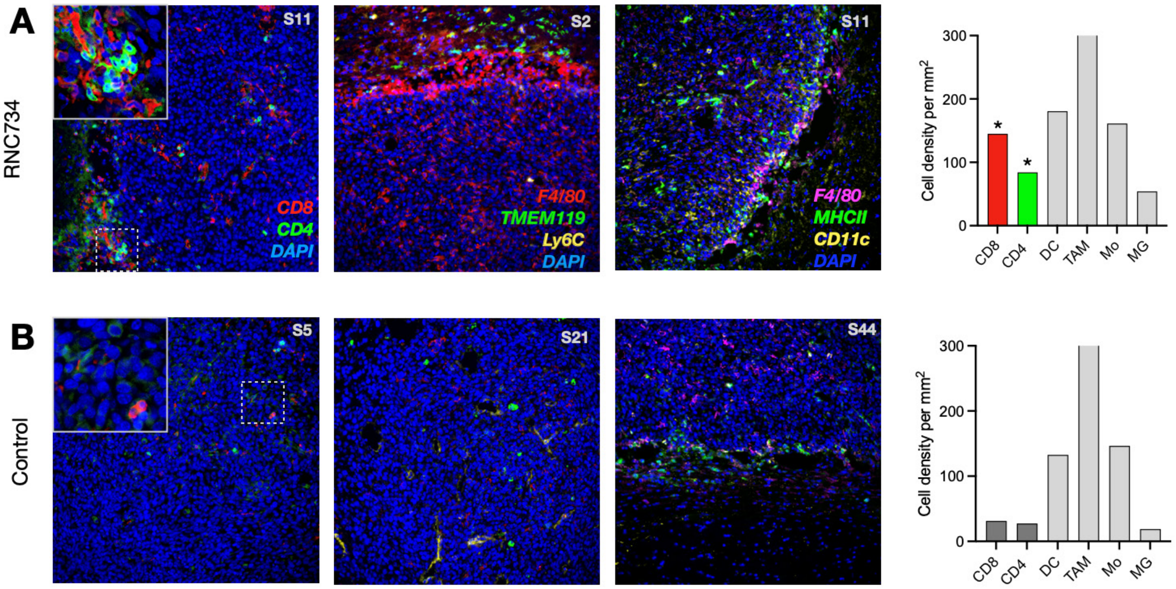

Figure. S11. In vivo IL12 induction by systemically administered RNC734
Intravital imaging of the CT2A tumor (CT2A-H2B-apple, red) microenvironment in IL12-eYFP reporter mice (green). Systemically administered RNC734 (white) is imaged 24 h after injection and seen in puncta with cells. The vast majority of RNC734-positive cells are induced to produce high amounts of IL12. 60x objective

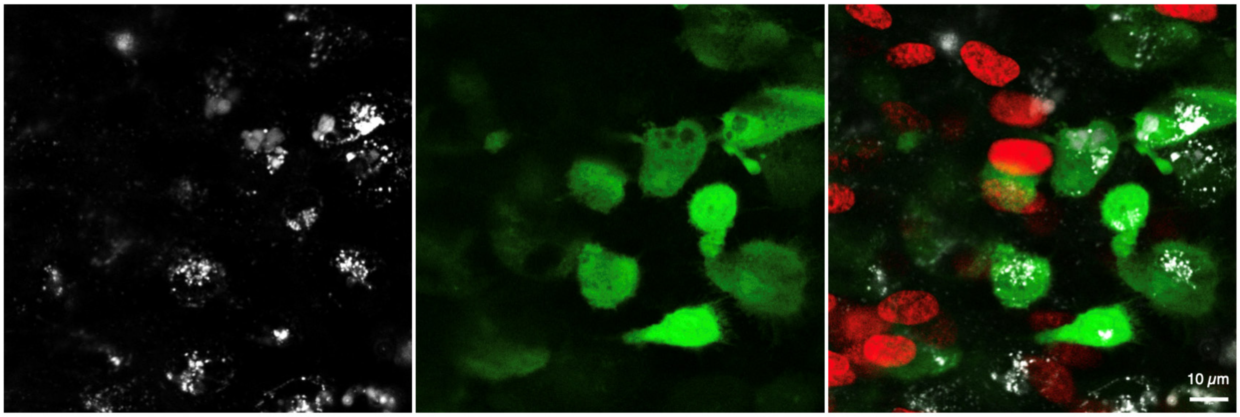

Figure. S12. Statistics on temporal IL12 induction in the CT2A glioblastoma model
Plotted are the number of IL12-eYFP positive cells per FOV in the TME of CT2A tumor bearing mice. RNC734 was administered on Day 0 and Day 8 (triangles). ****: p < 0.0001; **: p< 0.05. ns: not significant. Each data point represents a different FOV. N= 3 mice

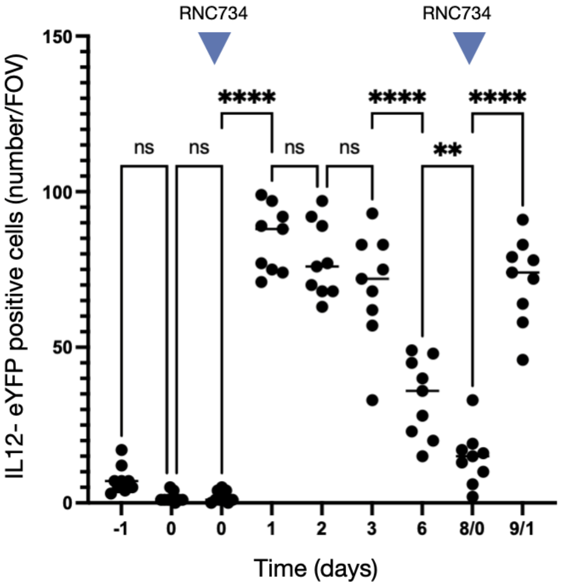

TABLES
